# Total knee arthroplasty using patient-specific instrumentation for osteoarthritis of the knee: a meta-analysis

**DOI:** 10.1186/s12891-019-2940-2

**Published:** 2019-11-23

**Authors:** Kazuha Kizaki, Ajaykumar Shanmugaraj, Fumiharu Yamashita, Nicole Simunovic, Andrew Duong, Vickas Khanna, Olufemi R. Ayeni

**Affiliations:** 1 Department of Health Research Methods, Evidence, and Impact, McMaster University Medical Centre, McMaster University, 1200 Main St W, Hamilton, Ontario L8N 3Z5 Canada; 2 Division of Orthopaedic Surgery, McMaster University, 1200 Main St W, Hamilton, Ontario L8N 3Z5 Canada; 3 Department of Orthopaedic surgery and rheumatology, Kyoto Shimogamo Hospital, 17 Shimogamo, Kyoto, 606-0866 Japan

**Keywords:** Total knee replacement, Total knee arthroplasty, Patient-specific instrumentation, Patient-matched instrumentation, Knee osteoarthritis, Patient-important outcome, Patient-reported outcome measure, Systematic review, Meta-analysis

## Abstract

**Background:**

Total knee arthroplasty using patient-specific instrumentation (TKA-PSI), which are disposable cutting block guides generated to fit each patient’s 3-dimensional knee anatomy, has been developed to treat patients with end-stage osteoarthritis of the knee. Surrogate markers such as radiographic malalignment have been well investigated, however, patient-important outcomes are not well examined to elucidate the efficacy of TKA-PSI. The aim of this review is to determine if TKA-PSI improves patient-reported outcome measures (PROM), surgery time, blood loss, transfusion and complications (e.g. surgical site infection, deep venous thrombosis, and revision TKA).

**Methods:**

We searched the Cochrane Central Register of Controlled Trials (CENTRAL), MEDLINE, EMBASE, and ongoing clinical trials. For PROMs, surgery time, blood loss, and transfusion rate, we included randomized controlled trials (RCT) comparing TKA-PSI and standard TKA to treat osteoarthritis of the knee. For complications, we also included non-randomized comparative studies (non-RCT).

**Results:**

This review includes 38 studies, 24 of which were RCT and 14 of which were non-RCT. These included a total of 3487 patients. The predominant population in the included studies highly reflected the general population, with 62% being female, aged over 60 and having end-stage osteoarthritis of the knee. TKA-PSI did not improve PROMs as compared to standard TKA for less than 1-year (mean difference 0.48, 95% confidence interval (CI) -1.92–0.97 in the Oxford knee score, mean 3-month follow-up) and for 1-year or more (mean difference 0.25, 95%CI − 4.39–4.89 in the WOMAC score, mean 29-month follow-up). TKA-PSI did not reduce surgery time (mean difference − 3.09 min, 95%CI -6.73–0.55). TKA-PSI decreased blood loss with a small effect size corresponding to a 0.4 g/dl hemoglobin decrease (95%CI 0.18–0.88), but did not decrease transfusion rate (risk difference − 0.04, 95%CI -0.09–0.01). TKA-PSI did not reduce complication rates (risk difference 0.00, 95%CI − 0.01–0.01 in the composite outcome).

**Conclusions:**

TKA-PSI does not improve patient-reported outcome measures, surgery time, and complication rates as compared to standard TKA. TKA-PSI decreases blood loss with a small effect, which is not enough to reduce transfusion rate.

## Background

Globally, the proportion of the aging population has been dramatically increasing [1]. Osteoarthritis (OA) of the knee is the most common joint disorder among people aged over 60. Among this population, 10% of males and 13% of females have symptomatic knee OA [2]. Total knee arthroplasty (TKA) is the most common surgical option for end-stage knee OA with knee deformity and persistent pain [3]. In the last 20 years (1993–2012), a total of 7.8 million primary TKA procedures were performed in the United States [4]. The number of TKA procedures continues to increase and it is expected to increase 69% between 2012 and 2050 [4]. Currently, TKA using intramedullary and extramedullary alignment systems with cutting guides is the standard of care [5]. During the procedure, numerous surgical devices are utilized to ensure success. However, this creates a complicated workflow and prolonged surgery time [6]. Furthermore, it requires positioning an intramedullary nail in the femoral canal that increases invasiveness.

A new innovative surgical technique using patient-specific instrumentation (PSI) for performing TKA has been developed to reduce the technical difficulties and invasiveness associated with standard TKA. PSI is also called “patient-matched instrumentation,” “custom-fit instrumentation,” or “custom-made instrumentation” [7–9]. For TKA using PSI, disposable cutting blocks are generated to fit each patient’s 3-dimensional anatomy of the knee in reference to the preoperative computed tomography (CT) or magnetic resonance imaging (MRI) images combined with radiographs of the lower extremity [10]. The cutting blocks are made individually for each patient and they enable the surgeon to develop a surgical plan specific to each patient. The cutting blocks fit on the distal femur and proximal tibia and guide surgeons to cut them accurately [10].

The TKA procedure using PSI decreases rates of lower limb malalignment [11, 12] and it is expected to improve functional outcomes and decrease revision rates [13–15]. In the procedure, PSI does not require positioning intramedullary nails in the femur and is expected to reduce blood loss and transfusion rates. Also, the simpler workflow owing to the cutting blocks potentially reduces surgical time. Long surgical time is one of the important risk factors for postoperative surgical site infection (SSI) [16] and deep venous thrombosis (DVT) [17] related to TKA. Thus, PSI is expected to reduce postoperative SSI and DVT.

Previous systematic reviews (SR) reported that TKA using PSI reduces surgical time, blood loss, and rates of lower limb malalignment as compared to standard TKA [12, 18–26]. However, the primary outcomes are surrogate markers and the SRs did not examine whether PSI would contribute to decreasing rates of transfusion, SSI, DVT, and revision TKA as compared to standard TKA. Also, the previous SRs addressed inconsistent results for patient-reported outcome measures (PROMs) with a limited number of RCTs. The aim of this review is to assess the effects of TKA using PSI for patients with osteoarthritis of the knee as compared to standard TKA. The complete PICO-format research questions is shown in Additional file 1: Appendix A.

## Methods

### Criteria for including studies

We included studies which compared TKA using PSI (TKA-PSI) and standard TKA in this review. Standard TKA was defined as TKA using intramedullary alignment guiding nail for cutting femur and either intramedullary or extramedullary alignment guiding nail for cutting tibia. Studies were included if patients had primary TKA for knee OA classified into grade 3 and 4, according to Kellgren-Lawrence grading system [27]. If patients had rheumatic diseases or if less than 80% of the study’s population had OA, the studies were excluded. Since rheumatic diseases are systematic diseases, controlling for these conditions using medications directly influences clinical outcomes. Types of primary outcome measures are shown below:

- PROMs such as Western Ontario and McMaster Universities Osteoarthritis Index (WOMAC), Oxford knee score (Oxford), Knee Society Score (KSS), and the Knee injury and Osteoarthritis Outcome Score (KOOS), European quality of life 5-dimensions using the visual analogue scale (EQ-5D VAS), 12-item short form health survey physical score (SF-12 physical score), 12-item short form health survey mental score (SF-12 mental score)
- Transfusion rate
- Blood loss
- Surgery time
- Complications (i.e. SSI, DVT, and revision TKA)

As a secondary outcome measure, we investigated the percentage of alignment outliers from the planned alignment in the included studies in which PROMs were examined.

For patient-reported outcome measures (PROM), surgery time, blood loss, and transfusion rate, we included randomized controlled trials (RCT). For complications (i.e. SSI, DVT, revision TKA), we also included non-randomized comparative studies (non-RCT).

We conducted a comprehensive literature search for all relevant articles using four electronic databases: the Cochrane Central Register of Controlled Trials (CENTRAL) (The Cochrane Library 2019, Issue 2), MEDLINE (1946 to February 15th, 2019), and EMBASE (1974 to February 15th, 2019). We also examined ongoing trials using the database of clinical trials (https://clinicaltrials.gov). Each searching strategies are shown in the Additional file 1: Appendix B. In hand-searching, we screened the reference list of previous SRs and relevant studies for additional articles potentially not identified through electronic search. We chose the key search terms “knee*,” “arthroplasty OR replacement,” and “patient-specific OR patient-matched OR custom-fit OR custom-made OR custom*.” The search was limited to articles published since 2001, because PSI was initially used in 2001 in institutional studies only, and the first report using PSI was published in 2004 [28].

We adopted a 3-step screening process (title screening, abstract screening, and full-text screening) to select eligible articles. After duplicate articles were removed, two reviewers (KK AND AS) independently performed title and abstract screenings. If either of the reviewers included an article during title or abstract screening, it was moved to the next stage for screening. During full-text screening, discrepancies were resolved through discussion and consensus with the senior authors (FY AND OA). We did not register this protocol in time before data collection was performed. For each study, two reviewers independently extracted data into a spreadsheet for the outcomes designed a priori. Differences were resolved by discussion.

Two reviewers independently assessed risk of bias for RCTs using the Cochrane risk of bias tool [29]. The following domains were assessed: sequence generation; allocation concealment; blinding of participants and personnel (performance bias); blinding of outcome assessment (separately for PROM, transfusion, blood loss, surgery time, and complications); incomplete outcome data (attrition bias); and selective reporting (reporting bias). For non-RCTs included for complications, we used the methodological index for non-randomized studies (MINORS) appraisal tool [30].

Dichotomous outcomes (transfusion and complications) were expressed as a risk ratio (RR) with 95% confidence intervals (CI). Continuous outcomes (i.e. PROM, blood loss, and surgery time) were expressed as a mean difference (MD) between TKA-PSI and standard TKA groups. We preferred to calculate effect size measures (standardized mean difference (SMD)), when studies used different instruments to assess the same outcome (e.g. blood loss).

We analyzed outcomes according to the modified intention-to-treat method without imputation. When data was not expressed with mean and standard deviation (SD), but expressed with median, minimum-maximum range, or interquartile range, we estimated the mean and SD in reference to Wan et al. (2014) [31].

Heterogeneity between pooled studies were assessed using the chi-square test with statistical significance set at *p* < 0.10 and the I^2^ statistic [32]. The I^2^ value was assessed as follows: 0–40% might not be important, 30–60% moderate, 50–90% substantial, and 75–100% considerable. Also, we considered variance of the point estimate and overlap in the confidence intervals.

For assessing reporting biases, we constructed funnel plots for each outcome for which there were at least five trials. We pooled included studies using the generic inverse variance method for continuous outcomes and the Mantel-Haenszel method for dichotomous outcomes in Review Manager 5 (Revman). Either one of fixed-effect or random- effect model was used depending on the heterogeneity. We created a ‘Summary of findings’ for the main comparison. For PROM, we selected an outcome measure in which most patients were pooled each for less than 1-year and for 1-year or more. We pre-specified and carried out sensitivity analyses by excluding non-RCTs in complications. We assessed the quality of evidence related to each outcome measure using the Grading of Recommendations Assessment, Development and Evaluation (GRADE) approach [33].

## Results

### Results of the search

**Fig. 1:**
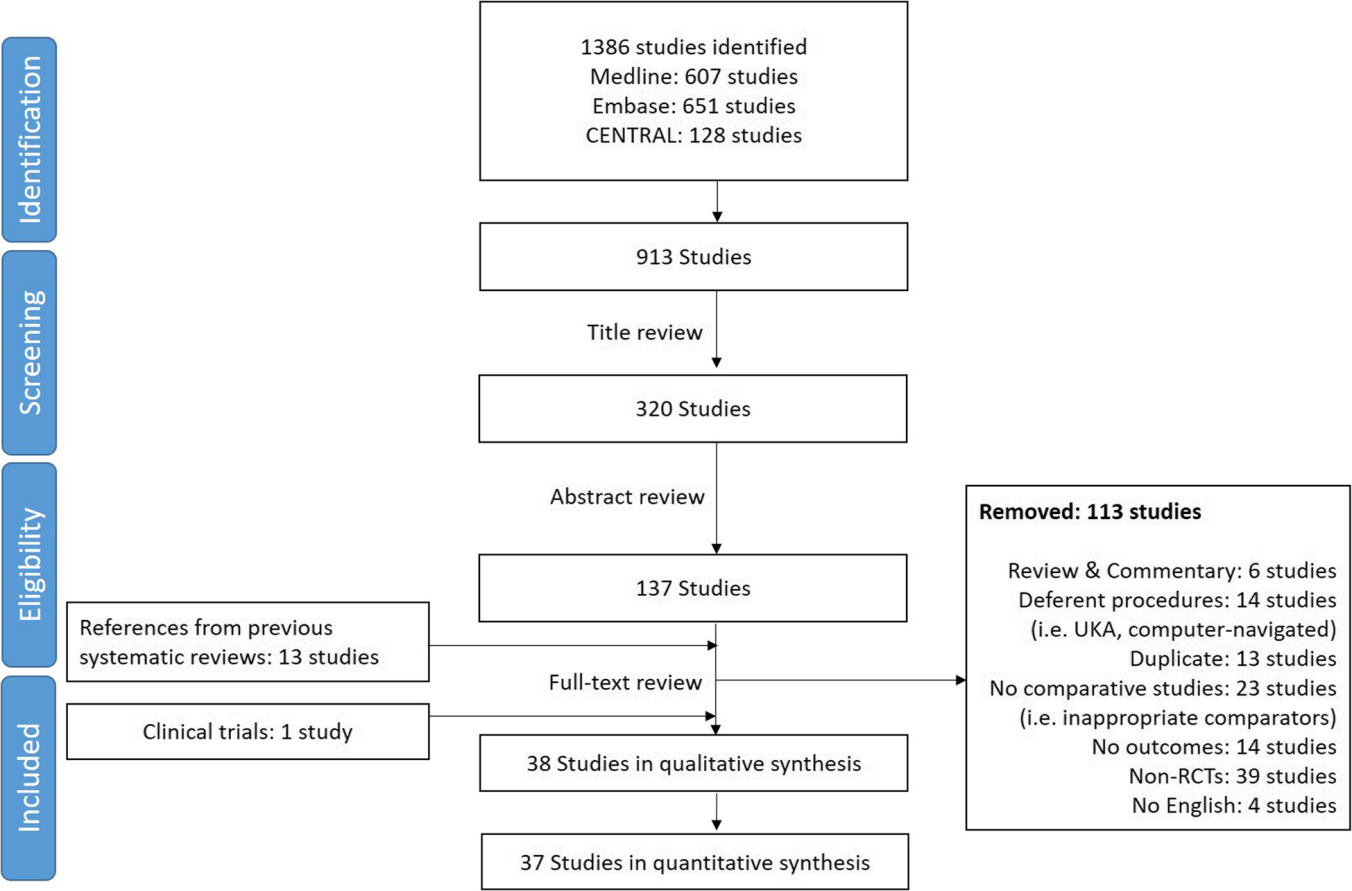
PRISMA flowchart

We searched from February 15th 2019 to March 15th 2019. We screened a total of 1386 articles (CENTRAL 128, EMBASE 651, MEDLINE 607). After the removal of duplicates, we checked 913 articles in title-screening and 320 articles in abstract-screening processes. We added 13 articles from the references in the pooled articles and implemented full-text screening process. From the database of clinical trials, we included 1 study. Overall, 38 articles were included in this systematic review: 38 studies were included in qualitative synthesis and 37 studies were included in quantitative synthesis (meta-analysis), as seen in Fig. 1. Details of the process of screening are illustrated in PRISMA (Preferred reporting items for systematic reviews and meta-analyses) flowchart in Fig. 1.

### Included studies

We included 38 studies, with a total of 3487 patients (1753 in TKA-PSI, and 1734 in standard TKA) [7, 34–71]. All studies except NCT02539992 were published in 2012–2018. We included 15 studies in PROM (14 studies in meta-analysis), 18 studies in surgery time (18 studies in meta-analysis), 15 studies in blood loss (15 studies in meta-analysis), 9 studies in transfusion rate (8 studies in meta-analysis), and 24 studies in complications (23 studies in meta-analysis). Boonen et al. (2013) and Boonen et al. (2016) were the same cohort and we counted them as the same study. Details of the included studies are provided in the characteristics of included studies table (Table 1)***.***

**Table 1:** Characteristics of included studies table

Author	Year	Country	Study design	Patients		%females		Age		Follow-up months	PSI device	CT/MRI	Eligibility criteria
				PSI	ST	PSI	ST	PSI	ST				
Noble Jr. [7]	2012	USA	RCT	15	14	47	57	65.4(57–76)	68.0(56–80)	NR	Visionaire	MRI	arthritis
Pietsch [34]	2013	Austria	RCT	40	40	68	53	71.4(6.6)	69.2(9.4)	3 months	Materialise	MRI	OA
Vundelinckx [35]	2013	Belgium	RCT	31	31	52	65	64.7(8.2)	68.2(8.5)	6 months	Visionaire	MRI	OA
Boonen [36]	2013	Netherlands	RCT	90	90	62	56	69.0(8.0)	65.0(8.8)	44 months	Signature	MRI	OA
Chareancholvanich [37]	2013	Thailand	RCT	40	40	85	90	69.5(55–84)	70.3(53–85)	NR	Materialise	MRI	OA
Hamilton [38]	2013	USA	RCT	26	26	46	73	68.1(52–86)	67.6(51–88)	NR	TruMatch	CT	OA
Roh [39]	2013	Korea	RCT	42	48	93	90	70.0(7.2)	70.0(5.1)	NR	Signature	CT	OA
Ng [40]	2014	USA	pros	51	27	67	52	65.6(7.5)	79.3(8.1)	3 months	Materialise	MRI	OA
Renson [41]	2014	Belgium	pros	71	60	62	52	68.2(8.9)	70.3(8.7)	6 months	Materialise	MRI	OA
Dossett [42]	2014	USA	RCT	44	44	7	14	66.0(7.7)	66.0(8.6)	24 months	Vanguard	MRI	arthritis
Abdel [43]	2014	France	RCT	20	20	60	60	71.0(61–81)	71(55–83)	3 months	Materialise	MRI	arthritis
Chotanaphuti [44]	2014	Thailand	RCT	40	40	88	88	69.7(5.5)	69.3(5.5)	1.5 months	TruMatch	CT	arthritis
Pfitzner [45]	2014	USA	RCT	60	30	57	57	64.0(54–74)	64.0(54–74)	3 months	Visionaire	MRI	OA
Woolson [46]	2014	USA	RCT	22	26	0	0	NR	NR	9 months	TruMatch	CT	no
Nabavi [47]	2015	Australia	retro	82	84	52	52	64(44–85)	65(45–88)	12 months	MyKnee	CT	no
Thienpont [48]	2015	Belgium	retro	75	75	67	67	65.6(9.6)	67.8(11.0)	1.5 months	Visionaire	NR	OA
Yan [49]	2015	China	RCT	30	30	57	80	67.5(8.0)	69.5(8.4)	3 months	Materialise	MRI	OA*
Chen [50]	2015	Singapore	pros	29	30	69	83	65.0(8.0)	65.0(8.0)	24 months	Materialise	MRI	OA
Kotela [51]	2015	Poland	RCT	49	46	67	72	66.1(8.4)	68.6(9.9)	12 months	Signature	CT	OA
Rathod [52]	2015	USA	retro	15	14	60	57	57.0(4.5)	59.0(6.5)	3 months	Visionaire	MRI	OA
Abane [53]	2015	France	RCT	70	70	43	39	67.8(47–84)	70.4(54–83)	3 months	Visionaire	MRI	OA
Molicnik [54]	2015	Slovenia	RCT	19	19	89	74	67.1(7.1)	66.8(6.7)	NR	Materialise	MRI	OA
Ferrara [55]	2015	Italy	RCT	15	15	60	53	75.3(6.7)	74.5(7.2)	NR	Materialise	MRI	OA
Anderl [56]	2016	Austria	pros	114	108	64	58	68.7(8.2)	67.7(9.6)	24 months	MyKnee	CT	OA
Boonen [57]	2016	Netherlands	RCT	90	90	62	56	69.0(8.0)	65.0(8.8)	44 months	Signature	MRI	OA
Huijbregts [58]	2016	Australia	RCT	69	64	58	50	66.7 (9.1)	69.0(9.6)	12 months	Visionaire	MRI	arthritis
Pourgiezis [59]	2016	Australia	pros	45	45	80	56	69.5(1.5)	69.3(1.5)	3 months	Visionaire	MRI	OA
White [60]	2016	USA	pros	21	42	67	67	59.1(7.4)	59.8(6.7)	24 months	iTotal	CT	OA
Culler [61]	2017	USA	retro	126	122	62	64	69.7(8.4)	68.3(9.5)	3 months	NR	CT	arthritis
Kwon [62]	2017	Korea	retro	48	50	96	94	73.0(4.3)	72.0(6.5)	3 months	Signature	MRI	OA
Zhu [63]	2017	Singapore	pros	42	48	71	77	69.3(7.2)	66.8(5.9)	24 months	TruMatch	CT	OA
Vide [64]	2017	Portugal	RCT	47	48	68	69	67.8(8.4)	69.3(6.5)	NR	Visionaire	MRI	OA
Kosse [65]	2018	Netherlands	RCT	21	21	62	43	62.7(4.5)	63.4(4.2)	12 months	Visionaire	MRI	OA
Steimle [66]	2018	USA	retro	31	49	NR	NR	60.8(8.5)	61.0(9.9)	NR	Materialise	MRI	OA
Tammachote [67]	2018	Thailand	RCT	54	54	78	72	72.0(7.0)	72.0(8.0)	24 months	Visionaire	MRI	arthritis
Maus [68]	2018	Germany	RCT	59	66	56	65	68.1(8.5)	71.5(8.1)	3 months	Imprint	MRI	OA
Stolarczyk [69]	2018	Poland	RCT	30	30	73	60	70.2(5.9)	69.6(7.1)	3 months	Visionaire	MRI	OA
Leeuwen [70]	2018	Norway	RCT	44	50	68	64	67.0(8.8)	64.0(6.9)	24 months	Signature	MRI	OA
NCT02539992 [71]	NR	Italy	RCT	26	18	38	44	NR	NR	12 months	Triathlon	MRI	OA

Date was expressed with mean (standard deviation). The underlined numbers represent minimum-maximum range. Abbreviations: *PSI* Patient-specific instrumentation, *ST* Standard TKA, *NR* Not reported, *RCT* Randomized controlled trial, *pros* Prospective non-RCT, *retro* Retrospective non-RCT, *OA* Osteoarthritis, *USA* The United States of America. Yan et al. (2015) recruited 93% OA and 6.7% RA

In the included studies, 29 studies with pooled 2547 patients explicitly included patients with osteoarthritis, whereas 7 studies with pooled 726 patients recruited patients with knee deformity. Twenty-seven studies used MRI-based PSI, whereas 10 studies used CT-based PSI. Many varieties of PSI device were used and the most prominent device was Visionnaire (Smith & Nephew, Memphis, USA) in 15 studies followed by Materialise (Zimmer via Materialise, Belgium) in 10 studies. Thirteen studies with the pooled 1384 patients followed for 1-year or more.

### Excluded studies

In full-text screening process, we excluded 113 studies. We described studies in which discrepancy for agreement was found or excluded with special reasons, being listed in Additional file 1: *Appendix C*. We found 18 ongoing trials using the database of *clinicaltrials.gov*, and we searched their results using corresponding author’s name and institution where trials were conducted. A list of ongoing trials was shown in Additional file 1: *Appendix D.*

### Risk of bias in included studies

We assessed the risk of bias in the included studies. In sequence generation, 8 out of 25 RCTs properly generated unpredictable randomization lists, creating low risk. In allocation concealment, 9 studies appropriately distanced from recruiters notifying allocation, whereas two studies were exposed to high risk of selection bias by recruiting participants in turns between PSI and standard TKA groups [35], or using block randomization without blinding block size for recruiters [65]. PSI requires preoperative MRI or CT to create patient-specific cutting blocks, and we assumed patients were notified as to whether they were allocated to the TKA-PSI group. For that reason, in almost all RCTs except for two studies, there was a high risk of bias in the domain of blinding of outcome assessment for PROM. In the two studies, all patients had preoperative MRI or CT and blinding of patients was robustly maintained with low risk of bias [42, 70]. Also, due to the trait of surgical trials, surgeons and scrub nurses could not be blinded. The unblinding potentially caused performance bias for surgery time, blood loss, and transfusion rate with high risk of bias. In incomplete outcome data, the following outcomes (i.e. surgery time, blood loss, transfusion rate) were less likely influenced, since these were perioperatively recorded in medical charts during admission. Six studies in PROM had high risk of attrition bias [42, 51, 53, 58, 68, 71]. In the included studies for the percentage of outliers in the positioning of the prosthetic implants, five studies clearly performed blinding to the outcome assessors for the images, having low risk of outcome detection bias [45, 53, 57, 58, 67].

In non-RCTs, the mean MINORS scores were 16.8 (SD 3.9). Seven studies were prospective non-RCTs, whereas 6 studies were retrospective non-RCTs. In only one study, the main study purpose was measuring complications. We presented the MINORS scores in the included non-RCTs in Additional file 1: *Appendix E.*

### Patient-reported outcome measures

The following outcome measures included two or more studies: KSS knee, KSS function, KSS total, Oxford, WOMAC, KOOS symptom, KOOS pain, KOOS ADL, KOOS sports, KOOS QoL, EQ-5D VAS, SF-12 physical score, and SF-12 mental score.

In patients followed for less than 1-year, 17 studies were included with the pooled 1609 patients. There were no significant differences in KSS knee, KSS function, KSS total, WOMAC, and Oxford scores between TKA-PSI and standard TKA (MD 0.24 (95%CI -2.25 – 3.65) in KSS knee at mean 3-months follow-up; MD 0.56 (95%CI -1.98 – 3.10) in KSS function at mean 3-months follow-up: MD -2.48 (95%CI -10.24 – 5.29) in KSS total at mean 3-months follow-up; MD 0.05 (95%CI -1.69 – 1.80) in WOMAC at mean 3-months follow-up, and MD -0.48 (95%CI -1.92 – 0.97) in Oxford score at mean 3-months follow-up). The MDs were less than minimally clinically important differences (MCID) in KSS knee (MCID 5.3), KSS function (MCID 6.1), WOMAC (MCID 10), and Oxford scores (MCID 4.3) [72–74]. There were no significant differences in EQ-5D VAS, SF-12 physical and mental scores between groups: MD -0.56 (95%CI -6.57 – 5.45) in EQ-5D VAS at mean 3-months follow-up; MD 0.51 (95%CI -2.58 – 3.59) in SF-12 physical score at mean 3-months follow-up (MCID 4.5); MD 1.84 (95%CI -3.02 – 6.69) in SF-12 mental score at mean 3-months follow-up (MCID 3.3) [72].

**Fig. 2:**
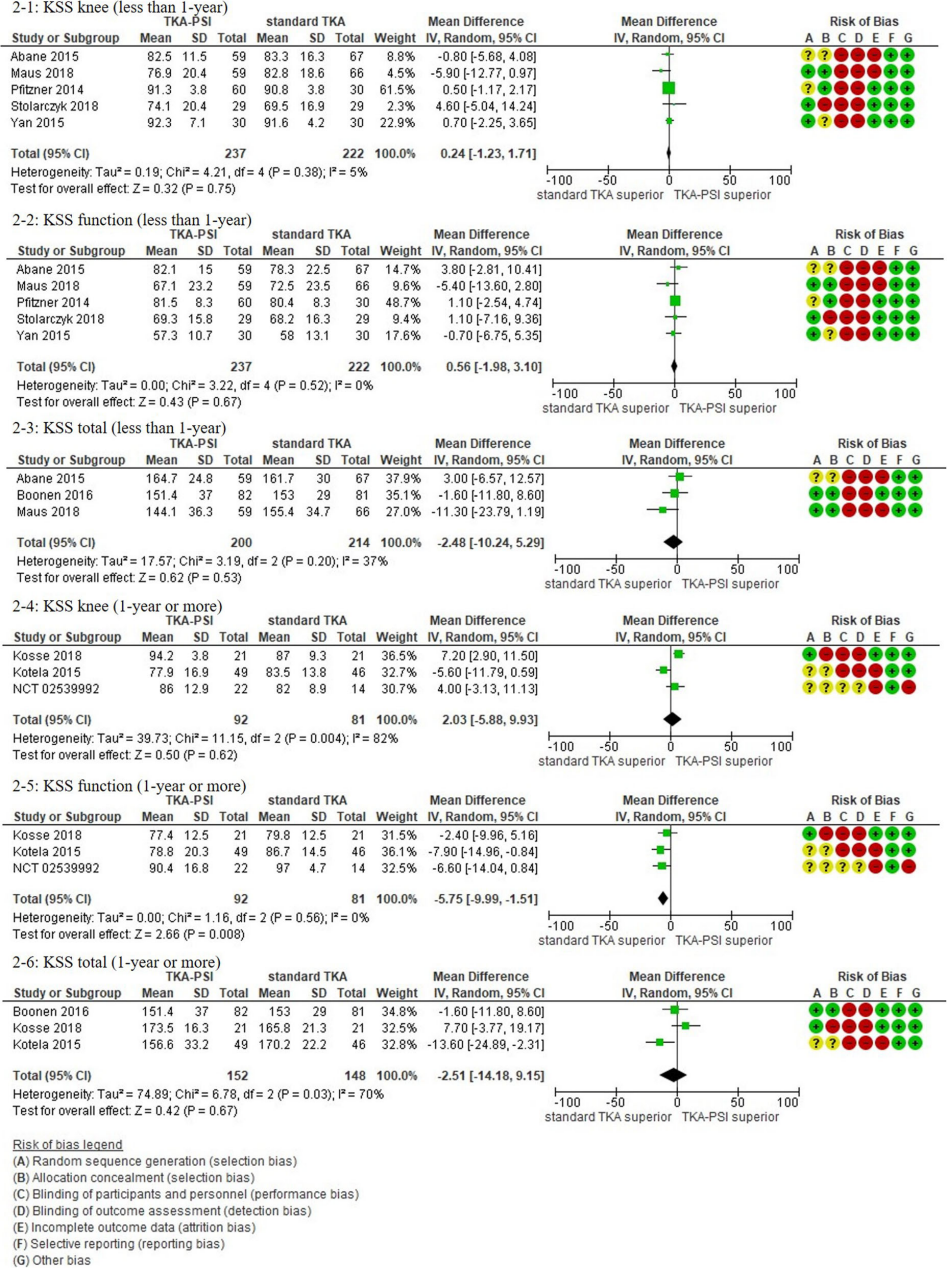
Forest plots in KSS

**Fig. 3:**
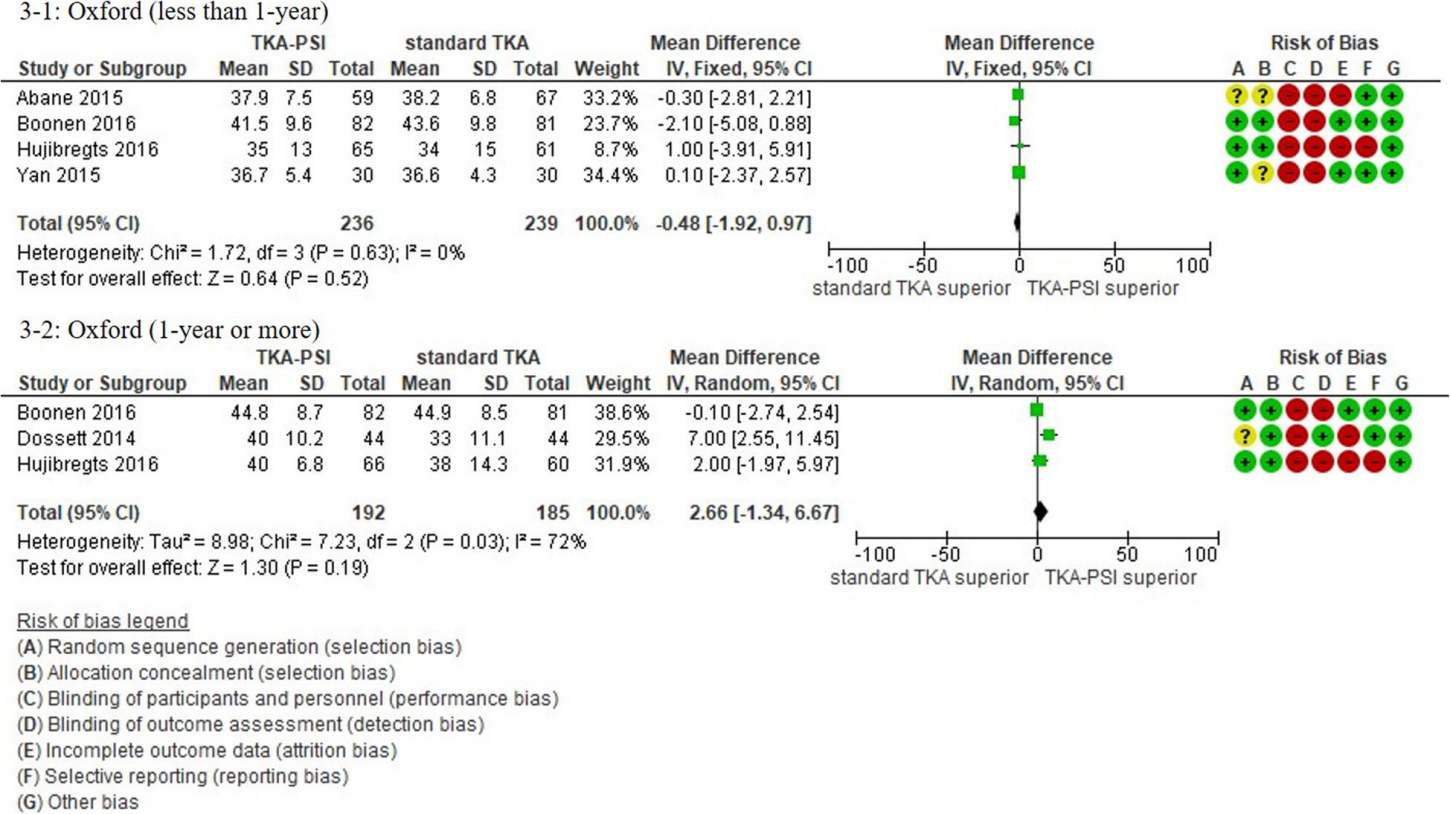
Forest plots in Oxford

**Fig. 4:**
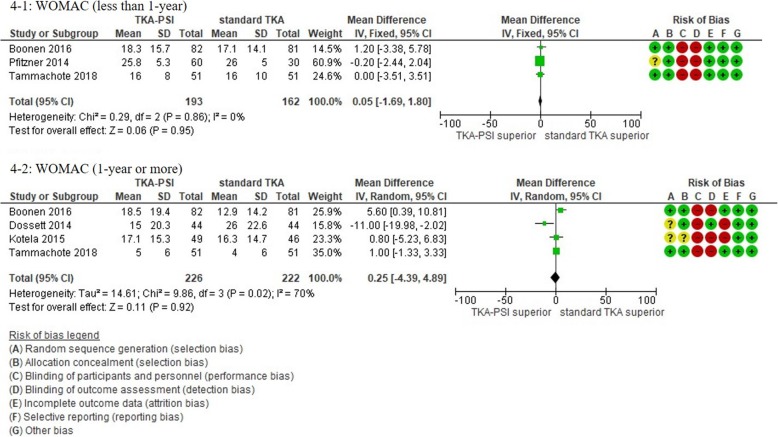
Forest plots in WOMAC

**Fig. 5:**
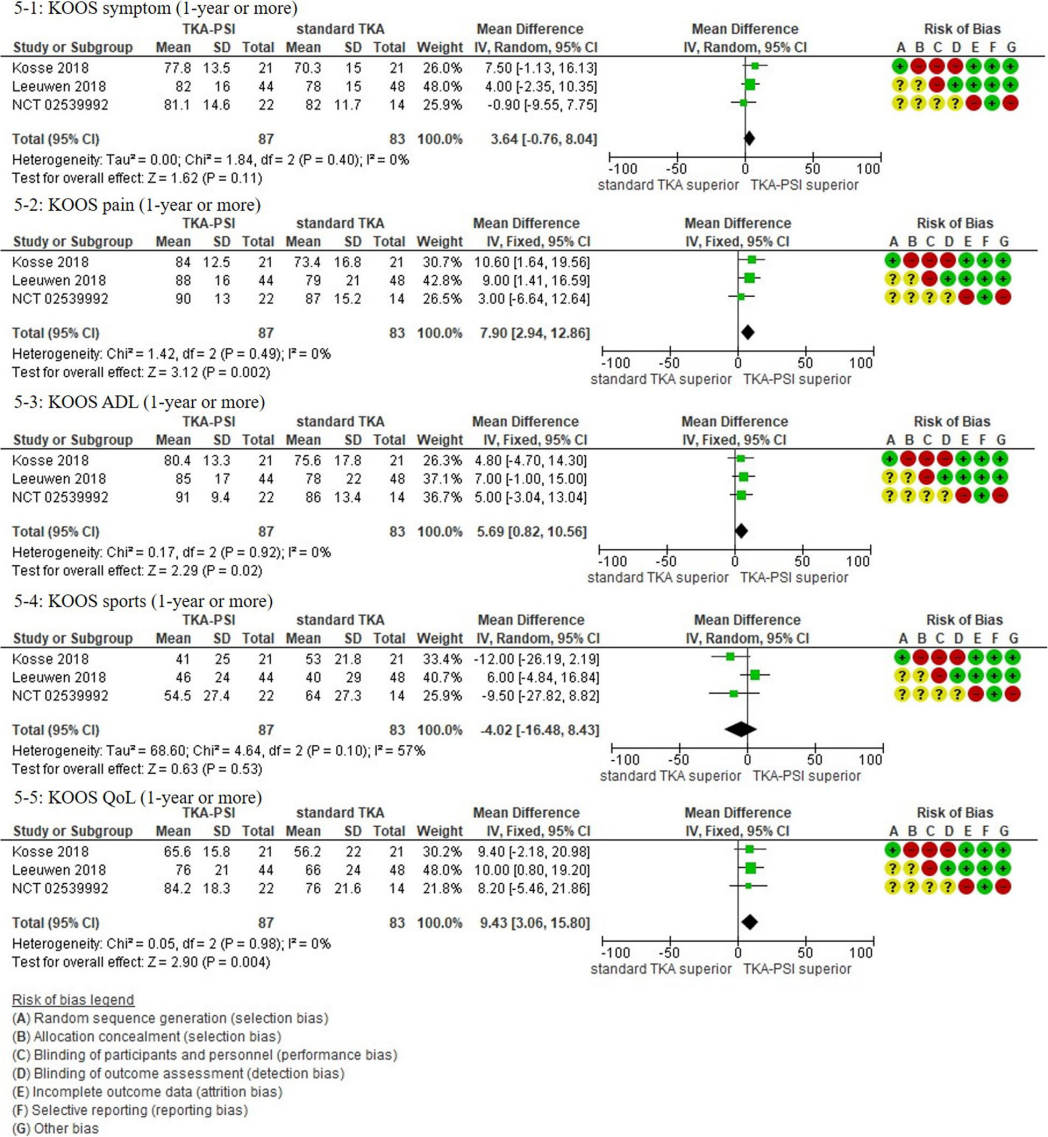
Forest plots in KOOS

**Fig. 6:**
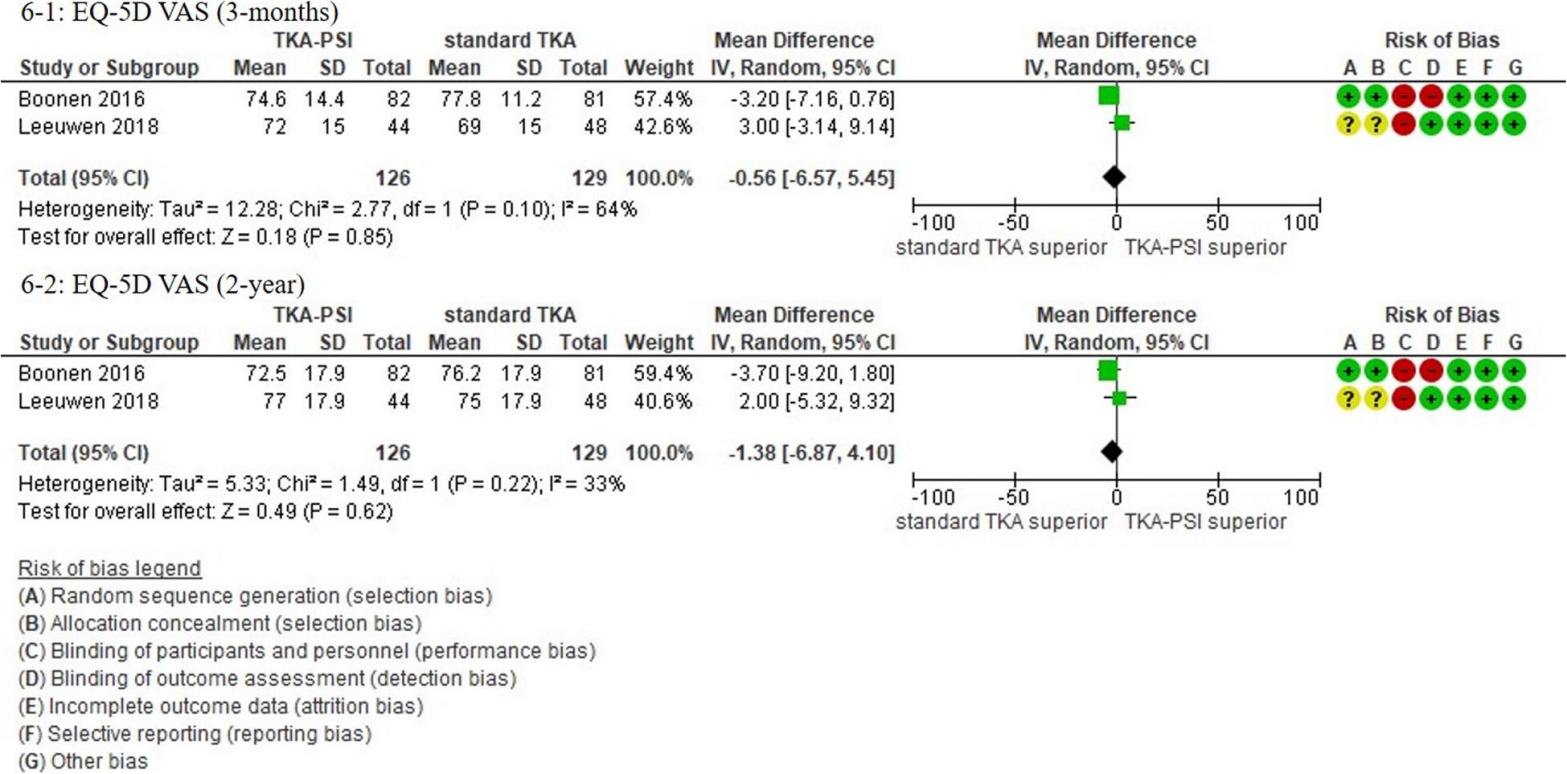
Forest plots in EQ-5D VAS

**Fig. 7:**
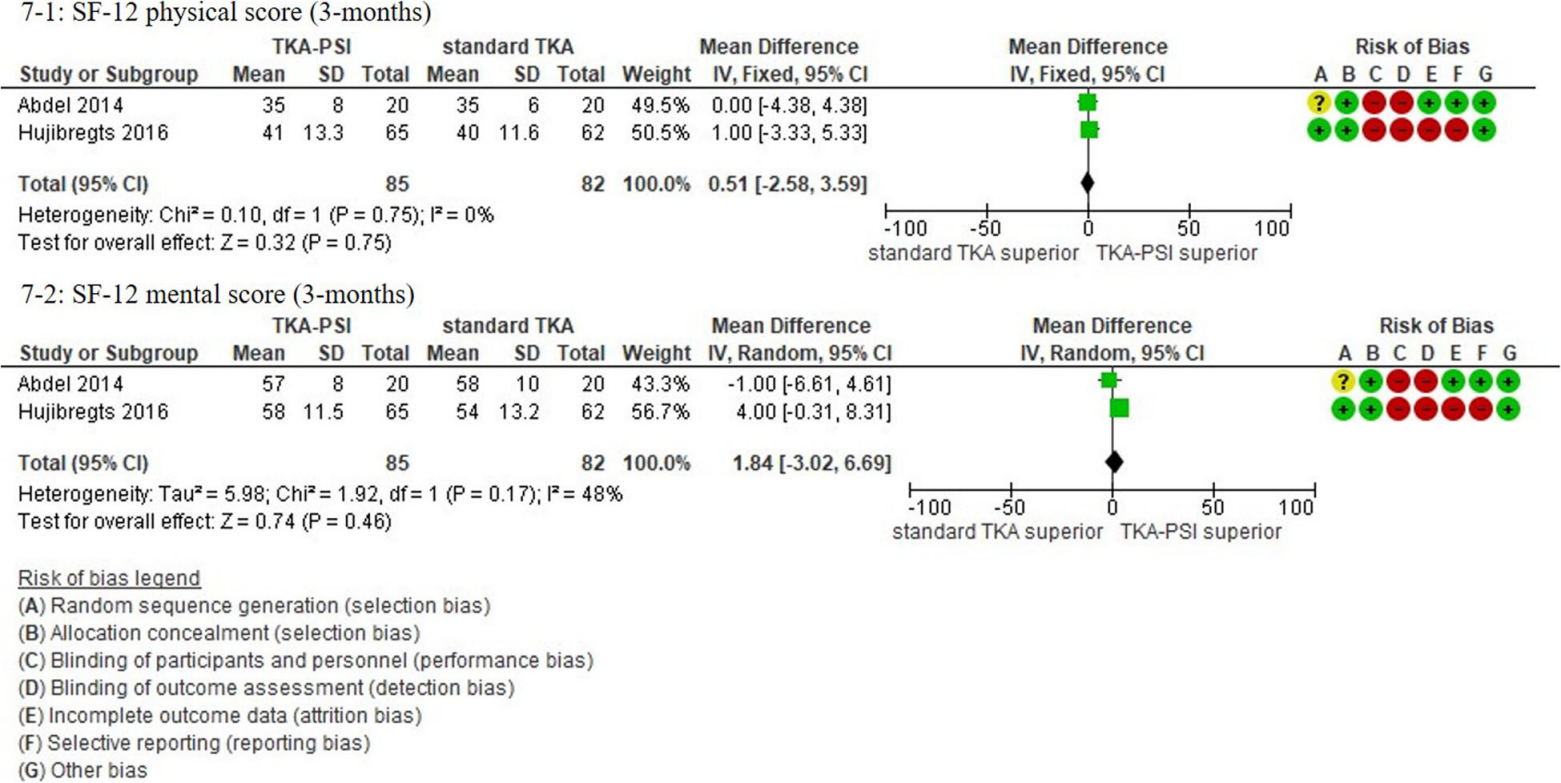
Forest plots in SF-12

In patients followed for 1-year or more, 13 studies were included with the pooled 1384 patients. There were no significant differences in Oxford, WOMAC, KSS knee and total, KOOS ADL and sports, and EQ-5D VAS between groups: MD 1.00 (95%CI -11.54 – 0.59) in KSS knee at 12-months follow-up, MD -5.22 (95%CI -10.70 – 0.06) in KSS function at 12-months follow-up, MD -2.51 (95%CI -14.18 – 9.15) in KSS total at mean 18.8-months follow-up, MD 2.66 (95%CI -1.34 – 6.67) in Oxford at mean 20.0-months follow-up, MD 0.25 (95%CI -4.39 – 4.89) in WOMAC at mean 21.6-months follow-up, MD 6.09 (95%CI 6.09–0.03 – 12.21) in KOOS ADL at mean 18.3-months follow-up, MD -2.39 (95%CI -19.99 – 15.20) in KOOS sports at mean 18.3-months follow-up, and MD -1.38 (95%CI -6.87 – 4.10) in EQ-5D VAS at mean 24-months follow-up). We found differences in KSS function, and KOOS symptom, pain, and QoL between groups, however, the pooled patients were small (less than 150) and the differences were less than the MCID: MD -5.34 (95CI -10.50 – − 0.18) in KSS function at mean 12-months follow-up (MCID 6.1), MD 5.23 (95%CI 0.11–10.35) in KOOS symptom at mean 18.3-months follow-up (MCID 10.7), MD 9.67 (95%CI 3.88–15.46) in KOOS pain at mean 18.3-months follow-up (MCID 16.7), MD 9.77 (95%CI 2.56–16.97) in KOOS QoL at mean 18.3-months follow-up (MCID 15.6) [73, 75]. We present more details in the forest plots in Figs. 2, 3, 4, 5, 6 and 7.

Two studies adopted unique outcome measures (i.e. Kujala [65] and Lysholm [35] scores), but they did not show differences in the outcome measures between TKA-PSI and standard TKA groups.

### Surgery time

**Fig. 8:**
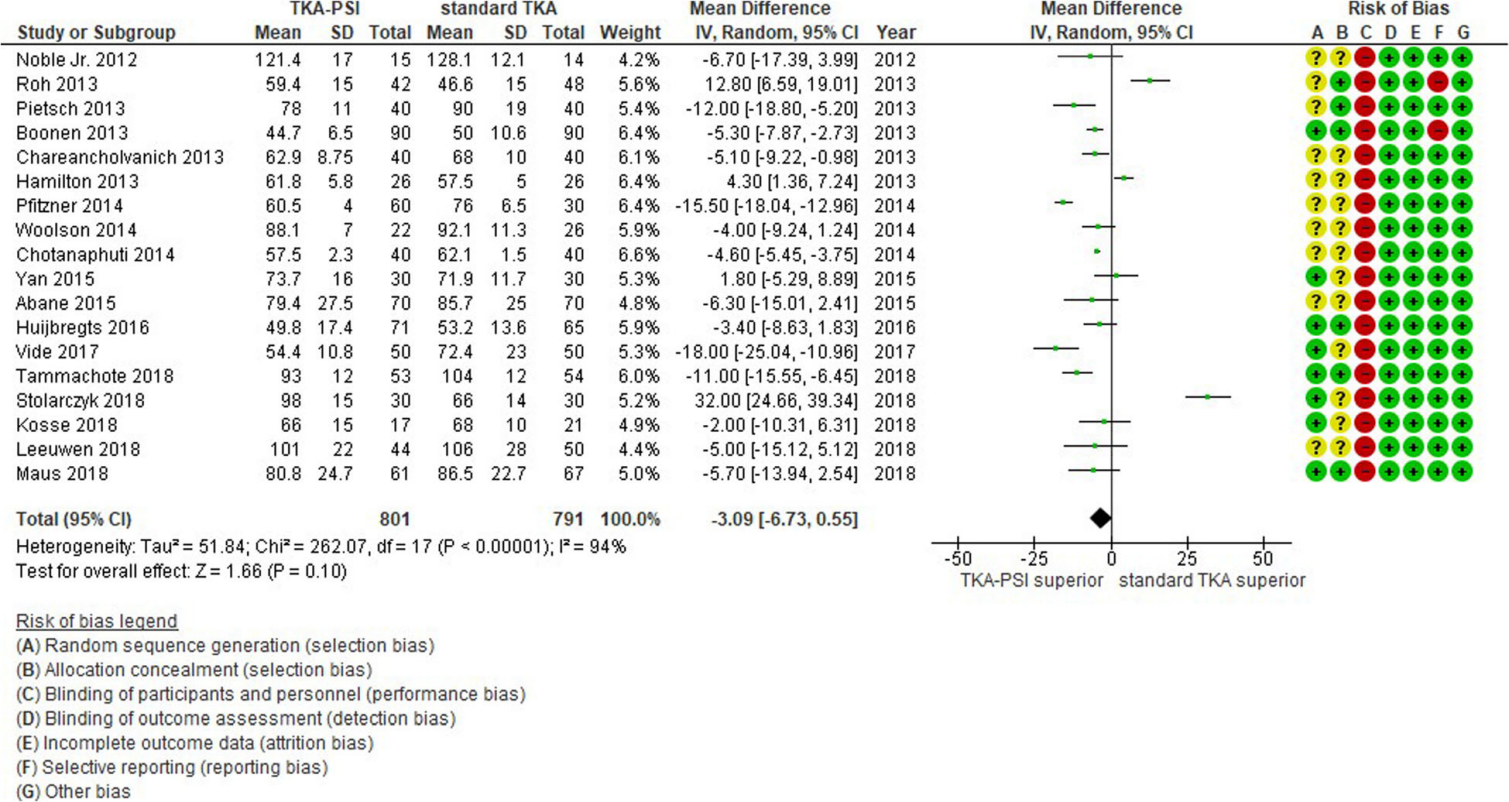
Forest plot in surgery time

Eighteen studies were included with the pooled 1592 patients. There was high heterogeneity between studies with I^2^ = 94%. The pooled mean difference was MD − 3.09 min (95%CI -6.73 – 0.55) and there was no significant difference between groups, as shown in Fig. 8.

### Blood loss

**Fig. 9:**
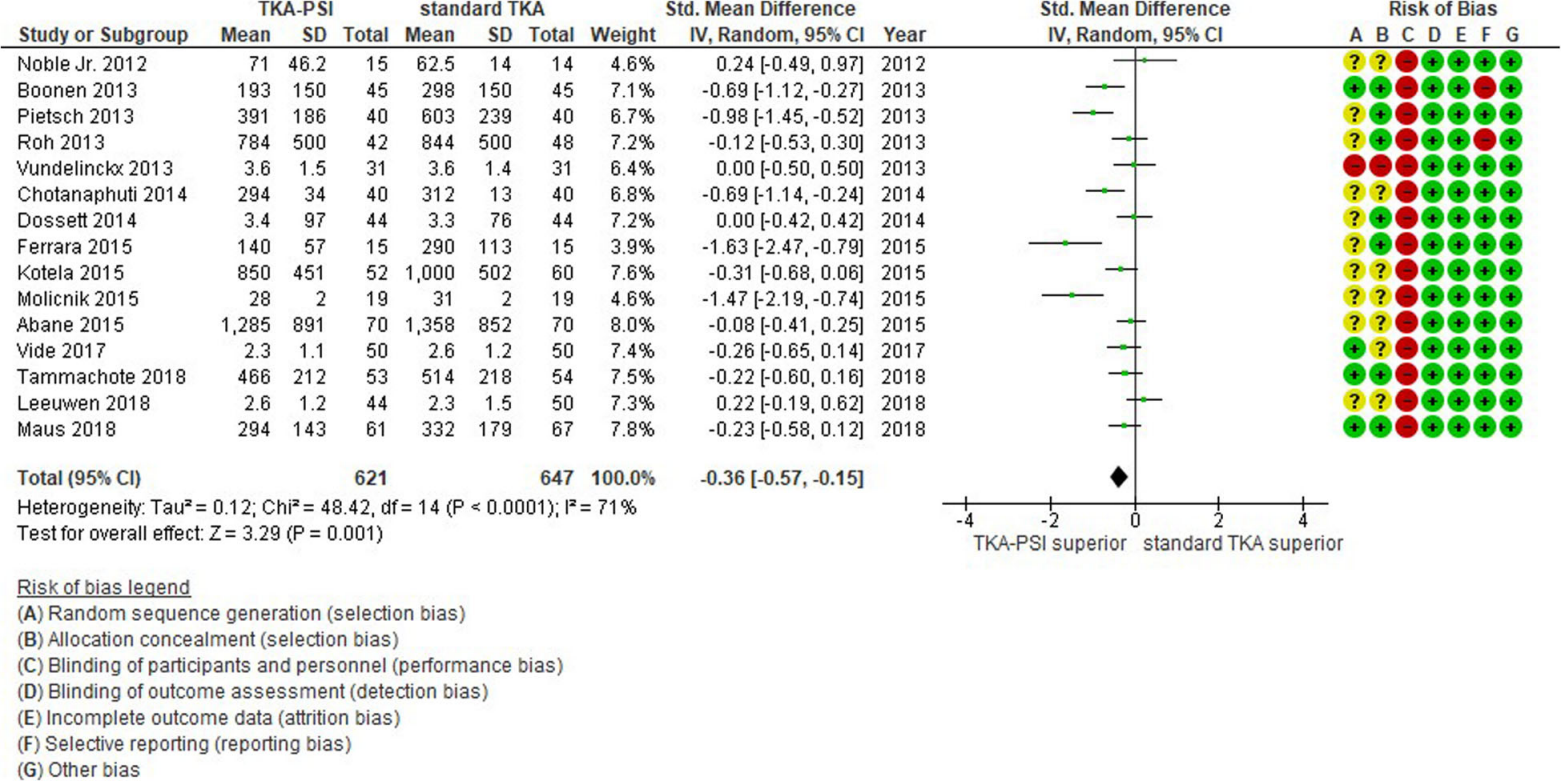
Forest plot in Blood loss (SMD)

Fifteen studies were included with various measuring scales and different time points when blood loss was measured postoperatively. In measuring blood loss, 5 studies measured volume of drainage fluid from suction drain device [34, 36, 39, 51, 67], 5 studies measured perioperative hemoglobin reduction [35, 42, 54, 64, 70], 3 studies measured intraoperative bleeding [7, 55, 68], 1 study calculated blood loss using the Mercuriali & Inghilleri formula [53, 76], and 1 study lacked information about the measurement [44]. The time points were most prominently at intraoperative in 3 studies, postoperative 24 h in 3 studies, 48 h in 2 studies, lowest hemoglobin in 1 study, and not shown in 4 studies. TKA-PSI decreased blood loss as compared to standard TKA with SMD -0.36 (95%CI -0.57 – − 0.15, *p* = 0.001) as seen in Fig. 9, although the effect size was small equivalent to hemoglobin 0.4 g/dl (95%CI 0.18–0.88) reduction. The effect size was estimated using the calculated SMD (− 0.36, 95%CI -0.57 - -0.15) and the pooled SD (1.2 g/dl) in hemoglobin decrease from a previous review examined blood loss after TKA [77, 78]: 0.43 g/dl (95%CI 0.18–0.88) = 0.36 (95%CI 0.15–0.57) × 1.2 g/dl.

### Transfusion rate

**Fig. 10:**
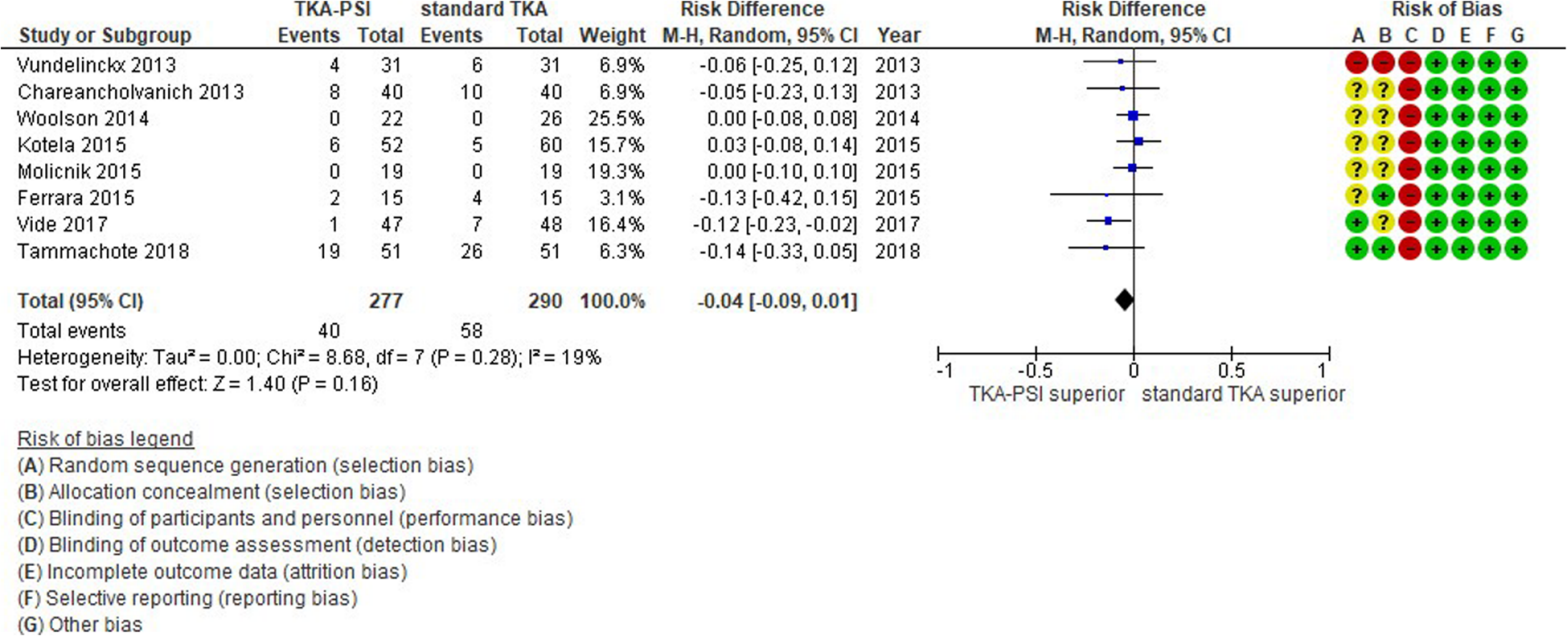
Forest plot in transfusion rate

Nine studies were chosen in qualitative synthesis and 8 studies were meta-analyzed with the pooled 487 patients. The overall transfusion rate was 17.3% (98/567), whereas two studies did not have any cases with transfusion. There was not significant difference in transfusion rate between TKA-PSI and standard TKA group with risk difference − 0.14 (95%CI -0.33 – 0.05, *p* = 0.16), as seen in Fig. 10. Pietsch et al. (2013) did not specify transfusion rate, but they described that there was no difference in transfusion rate in the trial.

### Complications

**Fig. 11:**
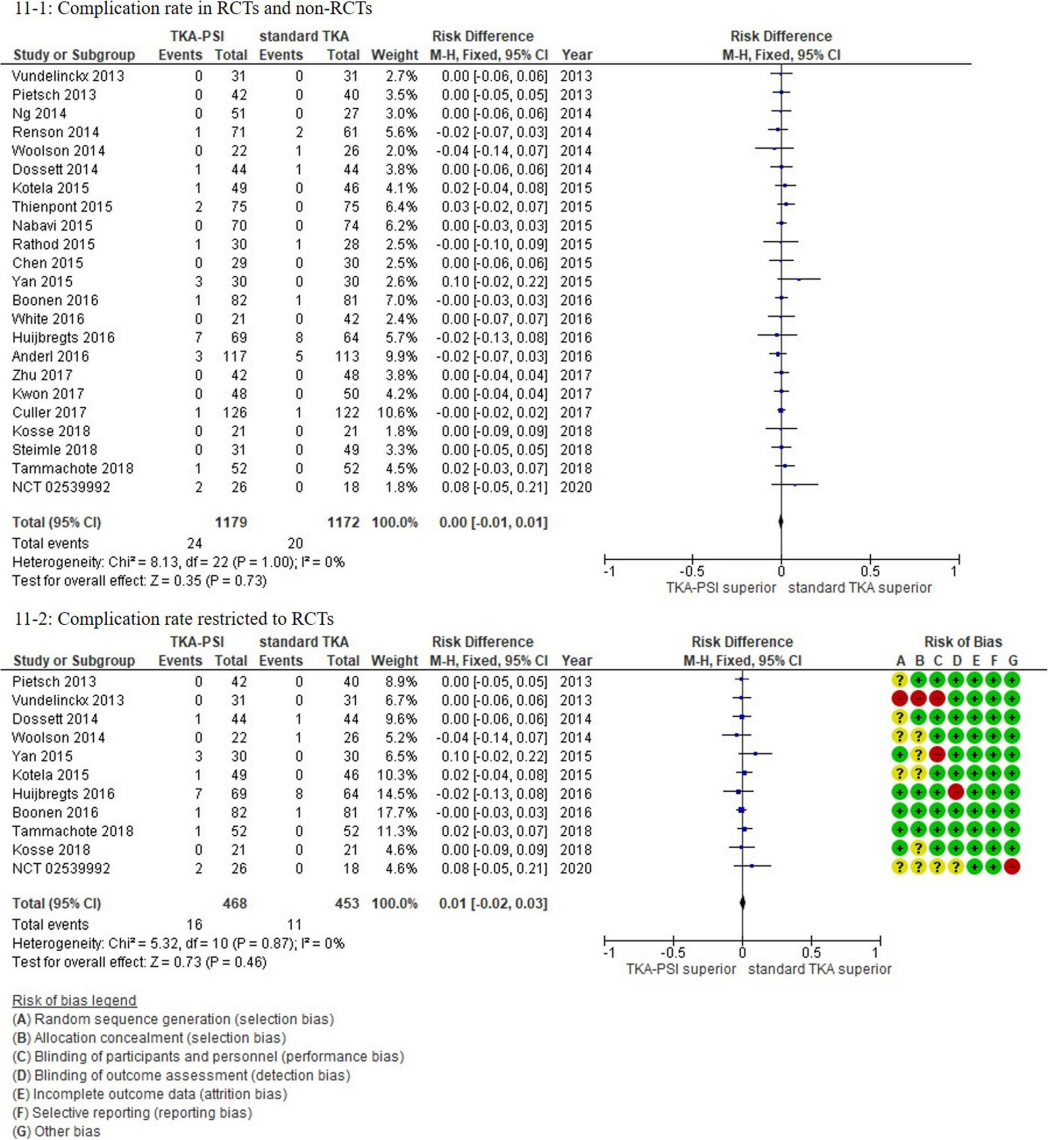
Forest plots in complication rate (composite outcome)

Overall, 24 studies were included with 11 RCTs and 13 non-RCTs. For SSI, 18 studies with 8 RCTs and 10 non-RCTs were included in meta-analysis. The incidence of SSI in the follow-up periods was 1.2% (24/2067). We measured a composite outcome consisting of SSI, DVT, and revision TKA. We did not find any difference in the composite outcome between TKA-PSI and standard TKA groups: risk difference 0.00 (95%CI -0.01 – 0.01, *p* = 0.73), as shown in Fig. 11*.* In sensitivity analysis excluding non-RCTs, the result was consistent: risk difference 0.01 (95%CI -0.02 – 0.03, *p* = 0.46).

There was no significant difference in SSI between TKA-PSI and standard TKA groups: 13 out of 1049 patient in TKA-PSI versus 11 out of 1018 patients in standard TKA with risk difference 0.00 (95%CI -0.01 – 0.01, *p* = 0.83) as seen in Additional file 1: *Appendix F*. In sensitivity analysis excluding non-RCTs, the result was consistent: risk difference 0.01 (95%CI -0.02 – 0.03, *p* = 0.59) in RCTs.

For DVT, 16 studies were included in meta-analysis with 7 RCTs and 9 non-RCTs. The incidence of DVT was 1.5% (12/1716) in the pooled trials. There was no significant difference between groups: 9 out of 881 patients in TKA-PSI versus 3 out of 835 in standard TKA with risk difference 0.01 (95%CI -0.01 – 0.02, *p* = 0.28) as seen in Additional file 1: *Appendix G*. In sensitivity analysis excluding non-RCTs, the result was consistent: risk difference 0.01 (95%CI -0.01 – 0.03, *p* = 0.44) in RCTs. Pulmonary emboli in Boonen et al. (2016) and NCT 02539992 were counted as DVT, considering the overlap between pulmonary embolism and DVT.

**Fig. 12:**
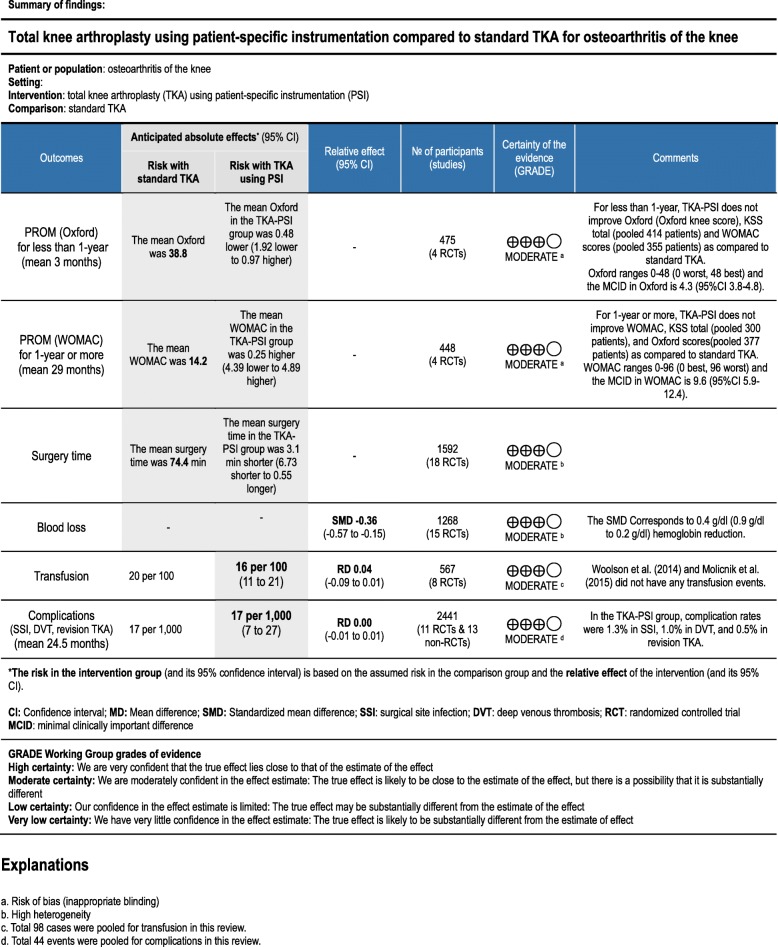
Summary of findings

For revision TKA, 14 studies were included and 13 studies were meta-analyzed with 4 RCTs and 9 non-RCTs. The incidence of revision TKA was 0.9% (11/1227) and there was no significant difference between groups: 3 out of 601 patients in TKA-PSI versus 8 out of 626 in standard TKA with risk difference − 0.01 (95%CI -0.02 – 0.01, *p* = 0.83) as seen in Additional file 1: *Appendix H*. The specific reasons for revision TKA were infection (*n* = 1), tibia loosening (*n* = 1), and patella resurfacing (*n* = 1) in TKA-PSI group, whereas infection (*n* = 2), tibia loosening (*n* = 2), patella resurfacing (*n* = 2), and instability (*n* = 2) in standard TKA group. Pourgiezis et al. (2016) experience one case with revision TKA in TKA-PSI group, but the data was not synthesized in meta-analysis, because the number of events in standard TKA was not shown. Also, Rathod et al. (2015) experienced reoperation for hematoma in TKA-PSI group, but we did not the case as revision TKA. Findings of the primary outcomes are summarized in ‘Summary of findings’ (Fig. 12).

### Lower-limb alignment (secondary outcome)

Eight studies were included with the pooled 930 patients [45, 49, 53, 57, 58, 67, 68, 70]. Lower-limb alignment was monitored using hip-knee-angle (HKA) [53, 58, 67, 70] and mechanical axis [45, 49, 57, 68]. The positioning of the femoral prosthetic implant was examined using coronal [45, 49, 53, 57, 58, 67, 70] and sagittal [49, 53, 57, 58, 70] radiographs or CT scans. Also, rotational alignment of the femoral prosthetic implant was examined using CT scans [45, 58, 70]. The positioning of the tibial prosthetic implant was examined using coronal [45, 49, 53, 57, 58, 67, 70] and sagittal [45, 49, 53, 57, 58, 70] radiographs or CT scans. In the included studies, rotational alignment of the tibial prosthetic implant was not examined. All studies defined outliers as three or more degrees deviation from the planned alignment.

There were no significant differences in HKA (95%CI -0.09 – 0.05, *p* = 0.58), mechanical axis (95%CI -0.21 – 0.16, *p* = 0.81), femoral coronal positioning (95%CI -0.05 – 0.05, *p* = 0.95), femoral sagittal positioning (95%CI -0.13 – 0.14, *p* = 0.91), femoral rotational positioning (95%CI -0.32 – 0.07, *p* = 0.21), tibial coronal positioning (95%CI -0.03 – 0.05, *p* = 0.60), and tibial sagittal positioning (95%CI -0.16 – 0.10, *p* = 0.63) when comparing between TKA-PSI and standard TKA, as shown in Additional file 1: Appendix *I*.

## Discussion

This systematic review included 38 studies (26 RCTs and 12 non-RCTs) to evaluate the efficacy of TKA using PSI as compared to standard TKA for patients with end-stage knee OA. For PROM, TKA-PSI did not show superior outcomes among patients followed for less than 1-year. Also, among patients followed for 1-year or more, we could not find clinically important differences between TKA-PSI and standard TKA groups. Lower-limb alignment and prosthetic implant positioning did not differ between TKA-PSI and standard TKA groups. TKA-PSI decreased perioperative blood loss, though the effect size was small. TKA-PSI did not reduce transfusion rate and surgery time. The most striking of this review was that we investigated three prominent complications (i.e. SSI, DVT, and revision TKA) and overall complication rates in TKA-PSI were small: 1.3% in SSI, 1.0% in DVT, and 0.5% in revision TKA in the short-term follow-up periods (maximum 44-months). We did not find any differences in complication rates between TKA-PSI and standard TKA groups, but the pooled events were insufficient to draw a conclusion.

We found 12 other systematic reviews assessing the efficacy of PSI as compared to standard TKA. Radiographic, CT-, or MRI-identified alignments of the components (i.e. mechanical axis) were the most common main outcomes in 9 reviews. Two reviews showed the efficacy of PSI for favorable mechanical axis and femoral rotational alignment. However, the effects of the favorable alignments on PROM and reduction of revision TKA were not reported in their reviews. Goyal et al. (2016) described that a meta-analysis with 5 RCTs showed no significant differences in PROMs between TKA-PSI and standard TKA, but not conclusive with limited numbers of the pooled patients (379 patients). Mannan et al. (2017) demonstrated that TKA-PSI did not improve PROM compared to standard TKA, including non-RCTs. In this review, we limited to RCTs and meta-analyzed the pooled 1299 patients (666 in TKA-PSI versus 633 in standard TKA) in PROM, concluding that PSI did not improve PROM among patients followed both for less than 1-year and for 1-year or more. The conclusion was consistent with the previous systematic reviews. Thienpont et al. (2017) reviewed that TKA-PSI decreased blood loss and surgery time; however, the potential benefits, such as decreasing transfusion, SSI and DVT, were not reported. In our review, TKA-PSI decreased blood loss with a small effect which corresponded to hemoglobin 0.4 g/dl (95%CI 0.1–0.7) reduction, but did not shorten surgery time. TKA-PSI did not reduce transfusion, SSI, and DVT rates.

The most noteworthy strength of this review is robust methodology: this review followed guidance from the Cochrane handbook of Systematic Reviews [79]. In our search of potentially eligible studies, we screened ongoing clinical trials. Also, we selected the main outcomes avoiding surrogate or interim outcomes [79]. Another strength is generalizability to the target population of interest. Among the included studies, 29 studies recruited patients solely diagnosed with knee OA. The prevalence of females in the pooled population was 62%, which highly reflected the general population [80]. Also, their ages were almost within 60–79, which is the predominant population in need of TKA [81]. Overall, the characteristics of the pooled population in this review accurately reflected the prevalence of knee OA in clinical practice. Also, 1361 patients were pooled in PROM and the generalizability of this review is favorable.

The most remarkable issue in the certainty of evidence was inappropriate blinding. Patients could not be blinded in the trait of PSI with preoperative MRI or CT. Also, surgeons could not be blinded, which potentially created performance bias. For these reasons, we interpreted the results in PROM as having a moderate quality of evidence. For surgery time and blood loss, each point estimate in the included studies varied with high heterogeneity (I^2^ = 94%), and we interpreted the evidence as being moderate in quality. The results in transfusion and complication rates were imprecise because of the small number of the pooled events with wide confidence intervals. We did not downgrade, but we suspected publication bias in the funnel plot in transfusion rate in Additional file 1: *Appendix J.* We interpreted them as having a moderate quality of evidence. The assessment sheet of quality of evidence for each outcome is presented in Additional file 1: *Appendix K.*

In limitation, we identified 4 studies in non-English literatures in the searching process, but we could not include the data in this review for our inability to literate the articles (language bias), as listed in Additional file 1: *Appendix C*. Also, in two studies, SD were not presented and we estimated the SD referring to the other studies in this review: Roh et al. (2013) and Boonen et al. (2013) for surgery time and blood loss. In this review, the longest follow-up periods were 44-month and future systematic reviews including studies with longer follow-up periods would be needed for examining long-term efficacy in addition to cost effectiveness using PSI. Also, a newer surgical device and procedure to perform robotic-arm assisted TKA, has been recently introduced with potentially superior clinical outcomes as compared to standard jig-based TKA [82]. Further studies are required to elucidate the full benefit and any limitations.

## Conclusions

TKA using PSI does not improve PROMs, surgery time and transfusion rate as compared to standard TKA among patients with end-stage OA of the knee followed for less than 1-year and for 1-year or more. TKA using PSI decreases blood loss with a small effect, but the effect is not enough to decrease transfusion rate. TKA using PSI does not reduce surgery time, and if it does, the degree of reduction is not clinically significant. TKA using PSI may not reduce SSI, DVT, and revision TKA, but they are inconclusive.

## Supplementary information

**Additional file 1: Appendix** ***A.*** *PICO* format research question. **Appendix B.1.** searching in Embase (1974 to February 15th, 2019). **Appendix B.2.** searching in Medline (1946 to February 15th, 2019). **Appendix B.3.** searching in CENTRAL (The Cochrane Library 2019, Issue 2). **Appendix C.** Excluded studies with special reasons. **Appendix D.** Unpublished ongoing studies. **Appendix E.** MINORS scores in non-RCTs. **Appendix F.1.** Forest plot in surgical site infection (RCTs and non-RCTs). **Appendix F.2**. Forest plot in surgical site infection (RCTs). **Appendix F.2**. Forest plot in surgical site infection (RCTs). **Appendix G.2**. Forest plot in DVT (RCTs). **Appendix G.2**. Forest plot in DVT (RCTs). **Appendix H.2**. Forest plot in revision TKA (RCTs). **Appendix I.1**. Forest plot in HKA. **Appendix I.2**. Forest plot in mechanical axis. **Appendix I.3**. Forest plot in femoral coronal alignment. **Appendix I.4**. Forest plot in femoral sagittal alignment. **Appendix I.5**. Forest plot in femoral rotational alignment. **Appendix I.6**. Forest plot in tibial coronal alignment. **Appendix I.7**. Forest plot in tibial sagittal alignment. **Appendix J**. Funnel plots. **Appendix K.1**. Assessment of the quality of the evidence for Oxford (less than 1-year). **Appendix K.2**. Assessment of the quality of the evidence for WOMAC (1-year or more). **Appendix K.3**. Assessment of the quality of the evidence for Surgery time. **Appendix K.4**. Assessment of the quality of the evidence for Blood loss. **Appendix K.5**. Assessment of the quality of the evidence for transfusion rate. **Appendix K.6**. Assessment of the quality of the evidence for complications.

## Data Availability

All data generated or analysed during this study are included in this published article and its supplementary information files.

## Abbreviations

ADL: Activities of daily living
CENTRAL: Cochrane Central Register of Controlled Trials
CI: Confidence interval
CT: Computed tomography
DVT: Deep venous thrombosis
EQ-5D VAS: European quality of life 5-dimensions using the visual analogue scale
GRADE: Grading of Recommendations Assessment, Development and Evaluation
HKA: Hip-knee-ankle angle
KOOS: Knee injury and Osteoarthritis Outcome Score
KSS: Knee Society Score
MCID: Minimally clinically important differences
MD: Mean difference
MINORS: Methodological index for non-randomized studies
MRI: Magnetic resonance imaging
OA: Osteoarthritis
Oxford: Oxford Knee Score
PRISMA: Preferred reporting items for systematic reviews and meta-analyses
PROM: Patient-reported outcome measure
PSI: Patient-specific instrumentation
QoL: Quality of life
RCT: Randomized controlled trial
RR: Risk ratio
SF-12 mental score: 12-item short form health survey mental score
SF-12 physical score: 12-item short form health survey physical score
SMD: Standardized mean difference
SR: Systematic review
SSI: Surgical site infection
TKA: Total knee arthroplasty
VAS: Visual analogue scale
WOMAC: Western Ontario and McMaster Universities Osteoarthritis Index

## References

[1] King AC, Guralnik JM (2010). Maximizing the potential of an aging population. JAMA.

[2] Zhang Y, Jordan JM (2010). Epidemiology of osteoarthritis. Clin Geriatr Med.

[3] Carr AJ, Robertsson O, Graves S, Price AJ, Arden NK, Judge A, Beard DJ (2012). Knee replacement. Lancet.

[4] Inacio MCS, Paxton EW, Graves SE, Namba RS, Nemes S (2017). Projected increase in total knee arthroplasty in the United States–an alternative projection model. Osteoarthr Cartil.

[5] Confalonieri N, Manzotti A, Pullen C, Ragone V (2005). Computer assisted technique versus intramedullary and extramedullary alignment systems in total knee replacement: a radiological comparison. Acta Orthop Belg.

[6] Sharkey PF, Hozack WJ, Rothman RH, Shastri S, Jacoby SM (2002). Why are total knee arthroplasties failing today?. Clin Orthop Relat Res.

[7] Noble JW, Moore CA, Liu N (2012). The value of patient-matched instrumentation in total knee arthroplasty. J Arthroplast.

[8] Klatt BA, Goyal N, Austin MS, Hozack WJ (2008). Custom-fit total knee arthroplasty (OtisKnee) results in malalignment. J Arthroplast.

[9] Bali K, Walker P, Bruce W (2012). Custom-fit total knee arthroplasty: our initial experience in 32 knees. J Arthroplast.

[10] Nam D, McArthur BA, Cross MB, Pearle AD, Mayman DJ, Haas SB (2012). Patient-specific instrumentation in total knee arthroplasty: a review. J knee Surg.

[11] Thienpont E, Schwab PE, Fennema P (2017). Efficacy of patient-specific instruments in total knee arthroplasty: a systematic review and meta-analysis. JBJS.

[12] Mannan A, Smith TO (2016). Favourable rotational alignment outcomes in PSI knee arthroplasty: a level 1 systematic review and meta-analysis. Knee.

[13] Huang NF, Dowsey MM, Ee E, Stoney JD, Babazadeh S, Choong PF (2012). Coronal alignment correlates with outcome after total knee arthroplasty: five-year follow-up of a randomized controlled trial. J Arthroplast.

[14] Longstaff LM, Sloan K, Stamp N, Scaddan M, Beaver R (2009). Good alignment after total knee arthroplasty leads to faster rehabilitation and better function. J Arthroplast.

[15] Fang DM, Ritter MA, Davis KE (2009). Coronal alignment in total knee arthroplasty: just how important is it?. J Arthroplast.

[16] Peersman G, Laskin R, Davis J, Peterson MGE, Richart T (2006). Prolonged operative time correlates with increased infection rate after total knee arthroplasty. HSS J.

[17] Fujita S, Hirota S, Oda T, Kato Y, Tsukamoto Y, Fuji T (2000). Deep venous thrombosis after total hip or total knee arthroplasty in patients in Japan. Clin Orthop Relat Res.

[18] Sassoon A, Nam D, Nunley R, Barrack R (2015). Systematic review of patient-specific instrumentation in total knee arthroplasty: new but not improved. Clin Orthop Relat Res.

[19] Thienpont E, Schwab PE, Fennema P (2014). A systematic review and meta-analysis of patient-specific instrumentation for improving alignment of the components in total knee replacement. Bone Joint J.

[20] Cavaignac E, Pailhe R, Laumond G, Murgier J, Reina N, Laffosse JM, Chiron P (2015). Evaluation of the accuracy of patient-specific cutting blocks for total knee arthroplasty: a meta-analysis. Int Orthop.

[21] Schotanus MG, Thijs E, Heijmans M, Vos R, Kort NP (2018). Favourable alignment outcomes with MRI-based patient-specific instruments in total knee arthroplasty. Knee Surg Sports Traumatol Arthrosc.

[22] Alcelik I, Blomfield M, Öztürk C, Soni A, Charity R, Acornley A (2017). A comparison of short term radiological alignment outcomes of the patient specific and standard instrumentation for primary total knee arthroplasty: a systematic review and meta-analysis. Acta Orthop Traumatol Turc.

[23] Mannan A, Akinyooye D, Hossain F (2017). A meta-analysis of functional outcomes in patient-specific instrumented knee arthroplasty. J knee Surg.

[24] Goyal T, Tripathy SK (2016). Does patient-specific instrumentations improve short-term functional outcomes after total knee arthroplasty? A systematic review and meta-analysis. J Arthroplast.

[25] Zhang QM, Chen JY, Li H, Chai W, Ni M, Zhang ZD, Yang F (2015). No evidence of superiority in reducing outliers of component alignment for patient-specific instrumentation for Total knee Arthroplasty: a systematic review. Orthop Surg.

[26] Fu H, Wang J, Zhou S, Cheng T, Zhang W, Wang Q, Zhang X (2015). No difference in mechanical alignment and femoral component placement between patient-specific instrumentation and conventional instrumentation in TKA. Knee Surg Sports Traumatol Arthrosc.

[27] Kellgren JH, Lawrence JS (1957). Radiological assessment of osteo-arthrosis. Ann Rheum Dis.

[28] Hafez MA, Chelule KL, Seedhom BB, Sherman KP (2004). Computer-assisted total knee replacement: could a two-piece custom template replace the complex conventional instrumentations?. Comput Aided Surg.

[29] Higgins JP, Altman DG, Gøtzsche PC, Jüni P, Moher D, Oxman AD (2011). The Cochrane Collaboration’s tool for assessing risk of bias in randomised trials. Bmj.

[30] Slim K, Nini E, Forestier D, Kwiatkowski F, Panis Y, Chipponi J (2003). Methodological index for non-randomized studies (MINORS): development and validation of a new instrument. ANZ J Surg.

[31] Wan X, Wang W, Liu J, Tong T (2014). Estimating the sample mean and standard deviation from the sample size, median, range and/or interquartile range. BMC Med Res Methodol.

[32] https://handbook-5-1.cochrane.org/chapter_9/9_5_2_identifying_and_measuring_heterogeneity.htm. Accessed 5 Jan 2019.

[33] Guyatt GH, Oxman AD, Vist GE, Kunz R, Falck-Ytter Y, Alonso-Coello P, Schünemann HJ (2008). GRADE: an emerging consensus on rating quality of evidence and strength of recommendations. Bmj.

[34] Pietsch M, Djahani O, Zweiger C, Plattner F, Radl R, Tschauner C, Hofmann S (2013). Custom-fit minimally invasive total knee arthroplasty: effect on blood loss and early clinical outcomes. Knee Surg Sports Traumatol Arthrosc.

[35] Vundelinckx BJ, Bruckers L, De Mulder K, De Schepper J, Van Esbroeck G (2013). Functional and radiographic short-term outcome evaluation of the Visionaire system, a patient-matched instrumentation system for total knee arthroplasty. J Arthroplast.

[36] Boonen B, Schotanus MGM, Kerens B, Van der Weegen W, van Drumpt RAM, Kort NP (2013). Intra-operative results and radiological outcome of conventional and patient-specific surgery in total knee arthroplasty: a multicentre, randomised controlled trial. Knee Surg Sports Traumatol Arthrosc.

[37] Chareancholvanich K, Narkbunnam R, Pornrattanamaneewong C (2013). A prospective randomised controlled study of patient-specific cutting guides compared with conventional instrumentation in total knee replacement. Bone Joint J.

[38] Hamilton WG, Parks NL, Saxena A (2013). Patient-specific instrumentation does not shorten surgical time: a prospective, randomized trial. J Arthroplast.

[39] Roh YW, Kim TW, Lee S, Seong SC, Lee MC (2013). Is TKA using patient-specific instruments comparable to conventional TKA? A randomized controlled study of one system. Clin Orthop Relat Res.

[40] Ng VY, Arnott L, Li J, Hopkins R, Lewis J, Sutphen S (2014). Comparison of custom to standard TKA instrumentation with computed tomography. Knee Surg Sports Traumatol Arthrosc.

[41] Renson L, Poilvache P, Van den Wyngaert H (2014). Improved alignment and operating room efficiency with patient-specific instrumentation for TKA. Knee.

[42] Dossett HG, Estrada NA, Swartz GJ, LeFevre GW, Kwasman BG (2014). A randomised controlled trial of kinematically and mechanically aligned total knee replacements: two-year clinical results. Bone Joint J.

[43] Abdel MP, Parratte S, Blanc G, Ollivier M, Pomero V, Viehweger E, Argenson JNA (2014). No benefit of patient-specific instrumentation in TKA on functional and gait outcomes: a randomized clinical trial. Clin Orthop Relat Res.

[44] Chotanaphuti T, Wangwittayakul V, Khuangsirikul S, Foojareonyos T (2014). The accuracy of component alignment in custom cutting blocks compared with conventional total knee arthroplasty instrumentation: prospective control trial. Knee.

[45] Pfitzner T, Abdel MP, von Roth P, Perka C, Hommel H (2014). Small improvements in mechanical axis alignment achieved with MRI versus CT-based patient-specific instruments in TKA: a randomized clinical trial. Clin Orthop Relat Res.

[46] Woolson ST, Harris AH, Wagner DW, Giori NJ (2014). Component alignment during total knee arthroplasty with use of standard or custom instrumentation: a randomized clinical trial using computed tomography for postoperative alignment measurement. JBJS.

[47] Nabavi A, Olwill CM (2015). Early outcome after total knee replacement using computed tomography–based patient-specific cutting blocks versus standard instrumentation. J Orthop Surg.

[48] Thienpont E, Grosu I, Paternostre F, Schwab PE, Yombi JC (2015). The use of patient-specific instruments does not reduce blood loss during minimally invasive total knee arthroplasty?. Knee Surg Sports Traumatol Arthrosc.

[49] Yan CH, Chiu KY, Ng FY, Chan PK, Fang CX (2015). Comparison between patient-specific instruments and conventional instruments and computer navigation in total knee arthroplasty: a randomized controlled trial. Knee Surg Sports Traumatol Arthrosc.

[50] Chen JY, Chin PL, Tay DKJ, Chia SL, Lo NN, Yeo SJ (2015). Functional outcome and quality of life after patient-specific instrumentation in total knee arthroplasty. J Arthroplast.

[51] Kotela A, Lorkowski J, Kucharzewski M, Wilk-Frańczuk M, Śliwiński Z, Frańczuk B (2015). Patient-specific CT-based instrumentation versus conventional instrumentation in total knee arthroplasty: a prospective randomized controlled study on clinical outcomes and in-hospital data. Biomed Res Int.

[52] Rathod PA, Deshmukh AJ, Cushner FD (2015). Reducing blood loss in bilateral total knee arthroplasty with patient-specific instrumentation. Orthopedic Clin.

[53] Abane L, Anract P, Boisgard S, Descamps S, Courpied JP, Hamadouche M (2015). A comparison of patient-specific and conventional instrumentation for total knee arthroplasty: a multicentre randomised controlled trial. Bone Joint J.

[54] Molicnik A, Naranda J, Dolinar D (2015). Patient-matched instruments versus standard instrumentation in total knee arthroplasty: a prospective randomized study. Wien Klin Wochenschr.

[55] Ferrara F, Cipriani A, Magarelli N, Rapisarda S, De Santis V, Burrofato A (2015). Implant positioning in TKA: comparison between conventional and patient-specific instrumentation. Orthopedics.

[56] Anderl W, Pauzenberger L, Kölblinger R, Kiesselbach G, Brandl G, Laky B (2016). Patient-specific instrumentation improved mechanical alignment, while early clinical outcome was comparable to conventional instrumentation in TKA. Knee Surg Sports Traumatol Arthrosc.

[57] Boonen B, Schotanus MGM, Kerens B, Van der Weegen W, Hoekstra HJ, Kort NP (2016). No difference in clinical outcome between patient-matched positioning guides and conventional instrumented total knee arthroplasty two years post-operatively: a multicentre, double-blind, randomised controlled trial. Bone Joint J.

[58] Huijbregts HJTAM, Khan RJK, Fick DP, Hall MJ, Punwar SA, Sorensen E (2016). Component alignment and clinical outcome following total knee arthroplasty: a randomised controlled trial comparing an intramedullary alignment system with patient-specific instrumentation. Bone Joint J.

[59] Pourgiezis N, Reddy SP, Nankivell M, Morrison G, VanEssen J (2016). Alignment and component position after patient-matched instrumentation versus conventional total knee arthroplasty. J Orthop Surg.

[60] White PB, Ranawat AS (2016). Patient-specific total knees demonstrate a higher manipulation rate compared to “off-the-shelf implants”. J Arthroplast.

[61] Culler SD, Martin GM, Swearingen A (2017). Comparison of adverse events rates and hospital cost between customized individually made implants and standard off-the-shelf implants for total knee arthroplasty. Arthroplasty Today.

[62] Kwon OR, Kang KT, Son J, Suh DS, Heo DB, Koh YG (2017). The effect of patient-specific instrumentation incorporating an extramedullary tibial guide on operative efficiency for total knee arthroplasty. Biomed Res Int.

[63] Zhu M, Chen JY, Chong HC, Yew AKS, Foo LSS, Chia SL (2017). Outcomes following total knee arthroplasty with CT-based patient-specific instrumentation. Knee Surg Sports Traumatol Arthrosc.

[64] Vide J, Freitas TP, Ramos A, Cruz H, Sousa JP (2017). Patient-specific instrumentation in total knee arthroplasty: simpler, faster and more accurate than standard instrumentation—a randomized controlled trial. Knee Surg Sports Traumatol Arthrosc.

[65] Kosse NM, Heesterbeek PJ, Schimmel JJ, van Hellemondt GG, Wymenga AB, Defoort KC (2018). Stability and alignment do not improve by using patient-specific instrumentation in total knee arthroplasty: a randomized controlled trial. Knee Surg Sports Traumatol Arthrosc.

[66] Steimle JA, Groover MT, Webb BA, Ceccarelli BJ (2018). Acute perioperative comparison of patient-specific instrumentation versus conventional instrumentation utilization during bilateral total knee arthroplasty. Surg Res Pract.

[67] Tammachote N, Panichkul P, Kanitnate S (2018). Comparison of customized cutting block and conventional cutting instrument in total knee arthroplasty: a randomized controlled trial. J Arthroplast.

[68] Maus U, Marques CJ, Scheunemann D, Lampe F, Lazovic D, Hommel H (2018). No improvement in reducing outliers in coronal axis alignment with patient-specific instrumentation. Knee Surg Sports Traumatol Arthrosc.

[69] Stolarczyk A, Nagraba L, Mitek T, Stolarczyk M, Deszczyński JM, Jakucinski M (2018). Does patient-specific instrumentation improve femoral and tibial component alignment in total knee arthroplasty? A prospective randomized study. Rehabilitation Science in Context.

[70] Van Leeuwen JA, Snorrason F, Röhrl SM (2018). No radiological and clinical advantages with patient-specific positioning guides in total knee replacement: a multicenter randomized controlled trial. Acta Orthop.

[71] Website on Clinicaltrials.govhttps://clinicaltrials.gov/ct2/show/NCT02539992. Accessed 15 Feb 2019.

[72] Clement ND, MacDonald D, Simpson AHRW (2014). The minimal clinically important difference in the Oxford knee score and short form 12 score after total knee arthroplasty. Knee Surg Sports Traumatol Arthrosc.

[73] Lee WC, Kwan YH, Chong HC, Yeo SJ (2017). The minimal clinically important difference for knee society clinical rating system after total knee arthroplasty for primary osteoarthritis. Knee Surg Sports Traumatol Arthrosc.

[74] Clement ND, Bardgett M, Weir D, Holland J, Gerrand C, Deehan DJ (2018). What is the minimum clinically important difference for the WOMAC index after TKA?. Clin Orthop Relat Res.

[75] Monticone M, Ferrante S, Salvaderi S, Motta L, Cerri C (2013). Responsiveness and minimal important changes for the knee injury and osteoarthritis outcome score in subjects undergoing rehabilitation after total knee arthroplasty. Am J Phys Med Rehab.

[76] Mercuriali F, Inghilleri G (1996). Proposal of an algorithm to help the choice of the best transfusion strategy. Curr Med Res Opin.

[77] Devji T, Johnston BC, Patrick DL, Bhandari M, Thabane L, Guyatt GH (2017). Presentation approaches for enhancing interpretability of patient-reported outcomes (PROs) in meta-analysis: a protocol for a systematic survey of Cochrane reviews. BMJ Open.

[78] Palmer A, Chen A, Matsumoto T, Murphy M, Price A (2018). Blood management in total knee arthroplasty: state-of-the-art review. J ISAKOS.

[79] Higgins JP, Green S (2008). Cochrane handbook for systematic reviews of interventions.

[80] Stundner O, Danninger T, Chiu YL, Sun X, Goodman SM, Russell LA (2014). Rheumatoid arthritis vs osteoarthritis in patients receiving total knee arthroplasty: perioperative outcomes. J Arthroplast.

[81] Kremers HM, Larson DR, Crowson CS, Kremers WK, Washington RE, Steiner CA, Jiranek WA, Berry DJ (2015). Prevalence of total hip and knee replacement in the United States. J Bone Joint Surg Am.

[82] Kayani B, Konan S, Tahmassebi J, Pietrzak JRT, Haddad FS (2018). Robotic-arm assisted total knee arthroplasty is associated with improved early functional recovery and reduced time to hospital discharge compared with conventional jig-based total knee arthroplasty: a prospective cohort study. Bone Joint J.

