# Supplementary material for: Total knee arthroplasty using patient-specific instrumentation for osteoarthritis of the knee: a meta-analysis
Source: BMC Musculoskelet Disord. 2019 Nov 23;20:561. doi: 10.1186/s12891-019-2940-2 (PMC6875166; doi:10.1186/s12891-019-2940-2)
Supplement: Supplementary file 1 — Additional file 1: Appendix A. PICO format research question. Appendix B.1. searching in Embase (1974 to February 15th, 2019). Appendix B.2. searching in Medline (1946 to February 15th, 2019). Appendix B.3. searching in CENTRAL (The Cochrane Library 2019, Issue 2). Appendix C. Excluded studies with special reasons. Appendix D. Unpublished ongoing studies. Appendix E. MINORS scores in non-RCTs. Appendix F.1. Forest plot in surgical site infection (RCTs and non-RCTs). Appendix F.2. Forest plot in surgical site infection (RCTs). Appendix F.2. Forest plot in surgical site infection (RCTs). Appendix G.2. Forest plot in DVT (RCTs). Appendix G.2. Forest plot in DVT (RCTs). Appendix H.2. Forest plot in revision TKA (RCTs). Appendix I.1. Forest plot in HKA. Appendix I.2. Forest plot in mechanical axis. Appendix I.3. Forest plot in femoral coronal alignment. Appendix I.4. Forest plot in femoral sagittal alignment. Appendix I.5. Forest plot in femoral rotational alignment. Appendix I.6. Forest plot in tibial coronal alignment. Appendix I.7. Forest plot in tibial sagittal alignment. Appendix J. Funnel plots. Appendix K.1. Assessment of the quality of the evidence for Oxford (less than 1-year). Appendix K.2. Assessment of the quality of the evidence for WOMAC (1-year or more). Appendix K.3. Assessment of the quality of the evidence for Surgery time. Appendix K.4. Assessment of the quality of the evidence for Blood loss. Appendix K.5. Assessment of the quality of the evidence for transfusion rate. Appendix K.6. Assessment of the quality of the evidence for complications. [file 12891_2019_2940_MOESM1_ESM.docx]

**Additional file**

**Appendix A: PICO format research question**

**P- Patients treated with total knee arthroplasty (TKA) for osteoarthritis of the knee**

**I- TKA using patient-specific instrumentation**

**C- Standard TKA**

**O- Patient-reported functional outcomes, transfusion rate, blood loss, surgery time, and complications (i.e. surgical site infection, deep venous thrombosis, and revision TKA)**

**Appendix B.1: searching in Embase (1974 to February 15^th^, 2019)**

--------------------------------------------------------------------------------

1 knee*.mp. (208519)

2 replacement arthroplasty/ or arthroplasty/ or total knee arthroplasty/ or knee arthroplasty/ or total

arthroplasty/ (38753)

3 replacement arthroplasty/ or knee replacement/ (3429)

4 2 or 3 (41078)

5 (patient-specific or patient-matched or custom-fit or custom-made or custom*).mp. [mp=title, abstract, heading

word, drug trade name, original title, device manufacturer, drug manufacturer, device trade name, keyword, floating

subheading word, candidate term word] (110498)

6 1 and 4 and 5 (676)

7 limit 6 to yr="2001 -Current" (651)

**Appendix B.2: searching in Medline (1946 to February 15^th^, 2019)**

--------------------------------------------------------------------------------

1 knee*.mp. (156518)

2 Arthroplasty, Replacement/ or Arthroplasty, Replacement, Knee/ or Arthroplasty/ (34435)

3 Arthroplasty, Replacement/ or Arthroplasty, Replacement, Knee/ (26371)

4 2 or 3 (34435)

5 (patient-specific or patient-matched or custom-fit or custom-made or custom*).mp. [mp=title, abstract, original

title, name of substance word, subject heading word, floating sub-heading word, keyword heading word, organism

supplementary concept word, protocol supplementary concept word, rare disease supplementary concept word, unique

identifier, synonyms] (76515)

6 1 and 4 and 5 (630)

7 limit 6 to yr="2001 -Current" (607)

**Appendix B.3: searching in CENTRAL (The Cochrane Library 2019, Issue 2)**

--------------------------------------------------------------------------------

1 (knee*):ti,ab,kw (20120)

2 (arthroplasty OR replacement):ti,ab,kw (30979)

3 (patient-specific OR patient-matched OR custom-fit OR custom-guide OR custom*):ti,ab,kw (4615)

4 1 and 2 and 3 (128)

**Appendix C: Excluded studies with special reasons**

| Author | Title | Reason |
| --- | --- | --- |
| Abane L. | Can a single-use and patient-specific instrumentation be reliably used in primary total knee arthroplasty? A multicenter controlled study | Compared with previously conducted TKA. |
| Chen J. | The radiological outcomes of patient-specific instrumentation versus conventional total knee arthroplasty | The same cohort as Chen et al. (2015) in Journal of Arthroplasty |
| Leon V. | Use of patient-specific cutting blocks reduces blood loss after total knee arthroplasty | In methods section, authors described they planned to monitor complications, but they did not show any data in result section. |
| Pourgiezis N. | Alignment and component position after patient-matched instrumentation versus conventional total knee arthroplasty | Complication rates in standard TKA group were not shown. |
| Nam D. | The impact of custom cutting guides on patient satisfaction and residual symptoms following total knee arthroplasty | In analysis, patients who had revision TKA in the follow-up periods were removed and we could not obtatin data for the patients. |
| Hintergrunde | Das Patientenspezifische MyKnee-Instrumentarium | no English |
| Schulze C. | OP-Zeit, Ergebnis und Ausbildungsstand des Chirurgen – der Einsatz patientenindividueller Instrumente bei der Implantation von Kniegelenkendoprothesen | no English |
| Sanz-ruiz P. | Moldes individualizados para alineación de la artroplastía primaria de rodilla | no English |
| Koster G. | Uni- und bikondylärer Oberflächenersatz | no English |

**Appendix D: Unpublished ongoing studies**

| Author | Institution | Protocol title |
| --- | --- | --- |
| Michael Dunbar | Dalhousie University | Application of Radiostereometric Analysis (RSA) to Triathlon Knee Components Inserted Using the OtisMed Custom-fit Total Knee Replacement System |
| David W. Manning | Northwestern University | Total Knee Replacement Component Alignment Using Manual Versus Custom Instrumentation |
| Washington University School of Medicine | Washington University School of Medicine | A Randomized Prospective Trial of Total Knee Arthroplasty Options Comparing Standard Knee Cutting Guides and MRI Generated Patient Specific Custom Cutting Guides |
| Douglas Naudie | Lawson Health Research Institute | Assessment of Conventional and Patient-specific Instrumentation in Total Knee Arthroplasty |
| Stephan M Rohrl | Oslo University Hospital | Patient-specific Positioning Guides (PSPG) Technique Versus Conventional Technique in Total Knee Arthroplasty |
| Zimmer Biomet | Zimmer Biomet | A Comparison Between Signature Total Knee Arthroplasty (TKA) to Conventional TKA and Computer Assisted TKA |
| Sheffield Teaching Hospitals NHS Foundation Trust | Sheffield Teaching Hospitals NHS Foundation Trust | Patient Specific Instrumentation in TKR |
| Yong In | The Catholic University of Korea | Conventional Versus Patient-specific Instruments in Total Knee Arthroplasty |
| Andrew Price | Oxford University Hospitals NHS Trust | The Assessment of Patient Specific Instrumentation for Unicompartmental Knee Replacement |
| Nantes University Hospital | Nantes University Hospital | Medico-economic Study of Patient Matched Cutting Blocks (Ancillary) MyKnee ® LBS During Knee Arthroplasty GMK ® |
| Smith & Nephew, Inc. | Smith & Nephew, Inc. | Visionaire™ Versus Standard Instrumentation Safety and Efficacy in Total Knee Arthroplasty (TKA) |
| Thomas Turgeon | Orthopaedic Innovation Centre | Custom Cutting Block Instrument vs Regular Instrumentation Total Knee Replacement (TKR) |
| Moussa Hamadouche | Cochin Hospital | Patient-Specific Versus Conventional Instrumentation in TKA |
| Smith & Nephew, Inc. | Smith & Nephew, Inc. | Axial Alignment in Patients Operated Using the Visionaire Patient Matched Cutting Blocks |
| Gavin Wood | Queen's University | TKA Using Patient-Specific Instrumentation |
| Medacta USA | Medacta USA | Single-use Efficiency Instruments With Patient Specific Technique (MyKnee®) Versus Traditional Metal Instruments With Conventional Surgical Technique |
| Nantes University Hospital | Nantes University Hospital | Medico-economic Interest of the Patient Matched Cutting Blocks (Ancillary) MyKnee ® LBS During the Poses of Complete Prosthesis of Knee GMK ®. Multicentric, Prospective, Controlled, Opened, Randomised Study About a Medical Device. (My Knee) |
| Smith & Nephew, Inc. | Smith & Nephew, Inc. | Visionaire Health Economics Study Comparing Economic Outcomes Between Visionaire and Standard Instrumentation |

**Appendix E: MINORS scores in non-RCTs**

|  | Anderl 2016 | Culler 2017 | Kwon 2017 | Nabavi 2015 | Ng 2014 | Pourgiezis 2016 | Steimle 2018 | Thienpont 2015 | Zhu 2017 | Chen 2015 | Rathod 2015 | Renson 2014 | White 115 |
| --- | --- | --- | --- | --- | --- | --- | --- | --- | --- | --- | --- | --- | --- |
| A clearly stated aim | 1 | 1 | 1 | 1 | 1 | 1 | 1 | 1 | 1 | 1 | 2 | 1 | 1 |
| Inclusion of consecutive patients | 2 | 0 | 2 | 0 | 2 | 2 | 2 | 2 | 2 | 2 | 2 | 2 | 2 |
| Prospective collection of data | 2 | 0 | 0 | 0 | 2 | 2 | 0 | 0 | 2 | 2 | 0 | 2 | 2 |
| Endpoints appropriate to the aim of the study | 2 | 1 | 1 | 1 | 1 | 1 | 1 | 1 | 1 | 1 | 1 | 1 | 1 |
| Unbiased assessment of the study endpoint | 2 | 2 | 2 | 2 | 2 | 2 | 2 | 2 | 2 | 2 | 2 | 2 | 2 |
| Follow-up period appropriate to the aim of the study | 2 | 0 | 0 | 2 | 0 | 0 | 0 | 2 | 2 | 2 | 2 | 2 | 2 |
| Loss to follow up less than 5% | 0 | 0 | 0 | 0 | 0 | 0 | 0 | 0 | 2 | 2 | 2 | 2 | 1 |
| Prospective calculation of the study | 2 | 0 | 0 | 0 | 2 | 0 | 0 | 0 | 2 | 2 | 0 | 0 | 0 |
| An adequate control group | 2 | 1 | 2 | 2 | 2 | 2 | 2 | 2 | 2 | 2 | 2 | 2 | 2 |
| Contemporary groups | 1 | 1 | 1 | 1 | 2 | 2 | 2 | 2 | 2 | 2 | 2 | 2 | 2 |
| Baseline equivalence of groups | 2 | 1 | 2 | 2 | 0 | 1 | 2 | 2 | 2 | 2 | 2 | 2 | 2 |
| Adequate statistical analysis | 2 | 2 | 2 | 2 | 2 | 2 | 2 | 2 | 2 | 2 | 2 | 2 | 2 |
| Total | 20 | 9 | 13 | 13 | 16 | 15 | 14 | 16 | 22 | 22 | 19 | 20 | 19 |

**Appendix F.1: Forest plot in surgical site infection (RCTs and non-RCTs)**


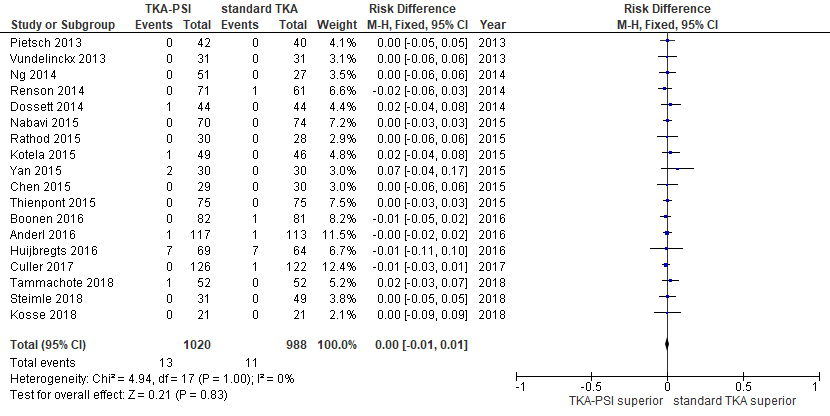


**Appendix F.2: Forest plot in surgical site infection (RCTs)**


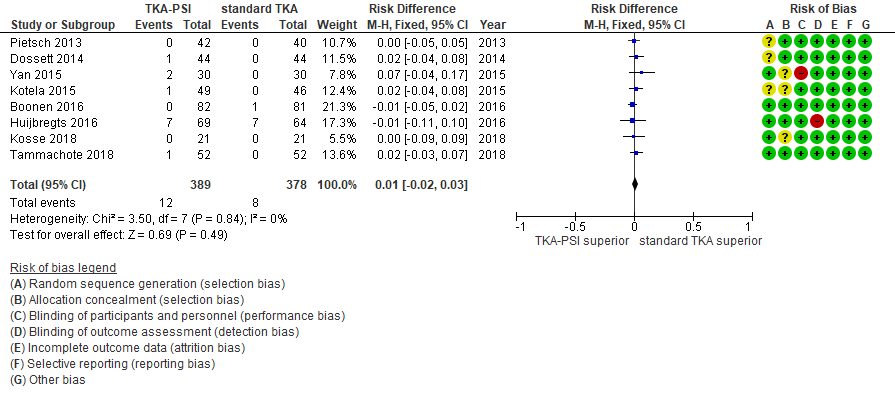


**Appendix G.1: Forest plot in DVT (RCTs and non-RCTs)**
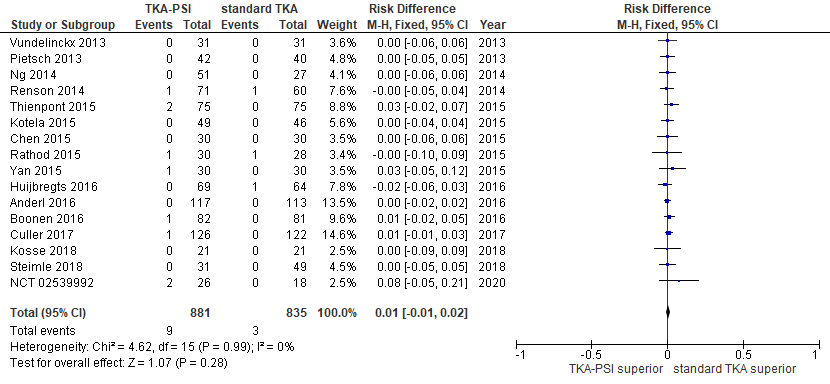


**Appendix G.2: Forest plot in DVT (RCTs)**


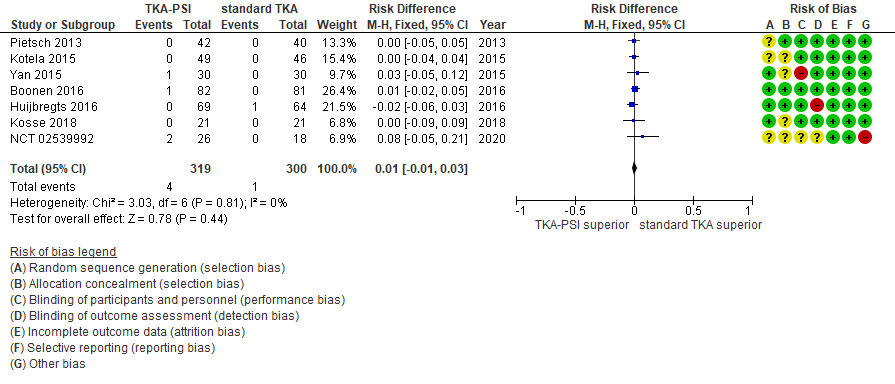


**Appendix H.1: Forest plot in revision TKA (RCTs and non-RCTs)**


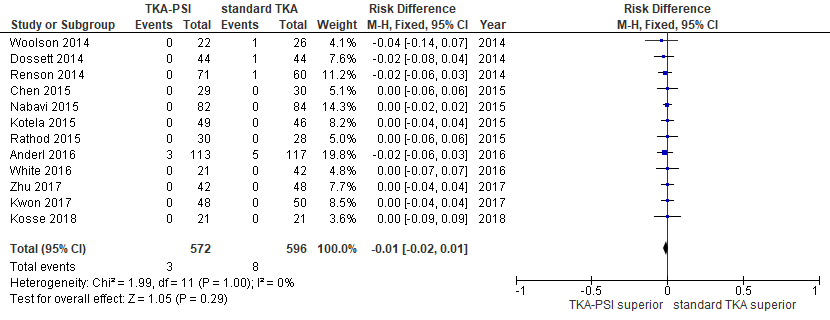


**Appendix H.2: Forest plot in revision TKA (RCTs)**


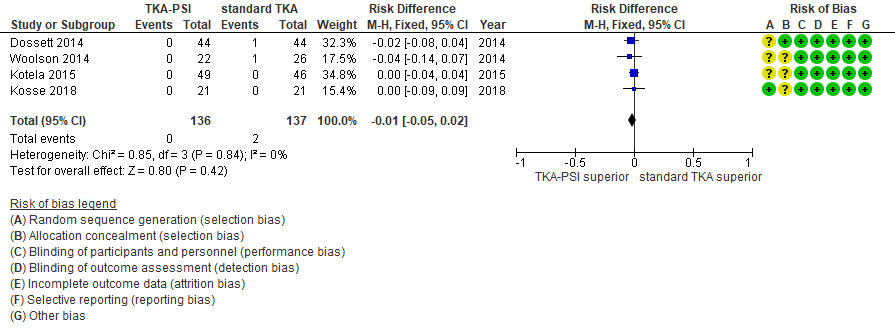


**Appendix I.1: Forest plot in HKA**


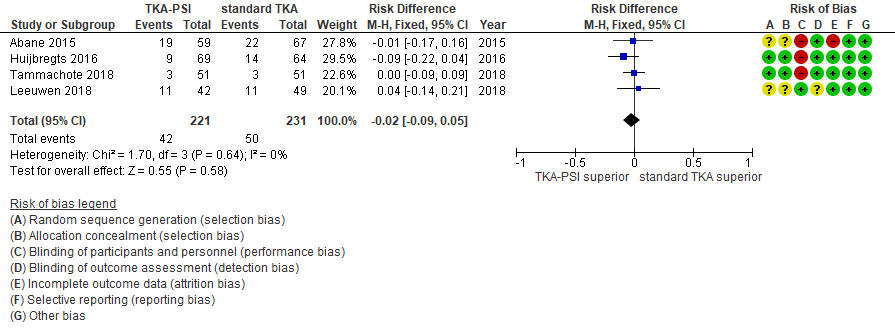


**Appendix I.2: Forest plot in mechanical axis**


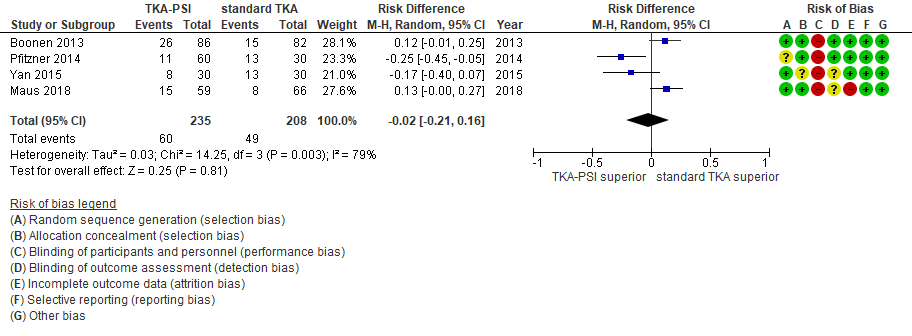


**Appendix I.3: Forest plot in femoral coronal alignment**


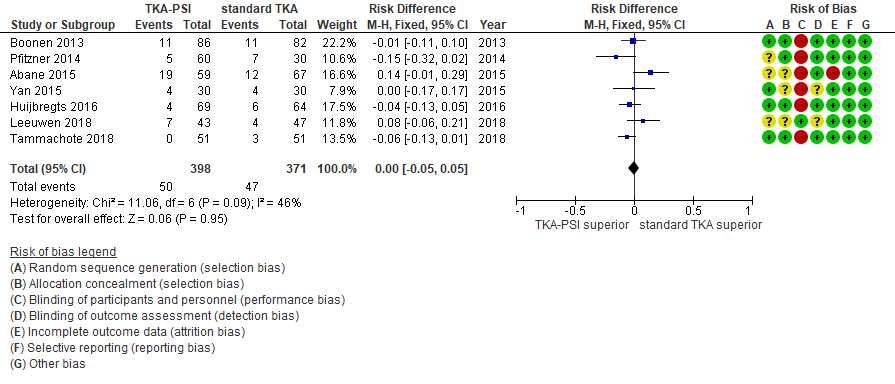


**Appendix I.4: Forest plot in femoral sagittal alignment**


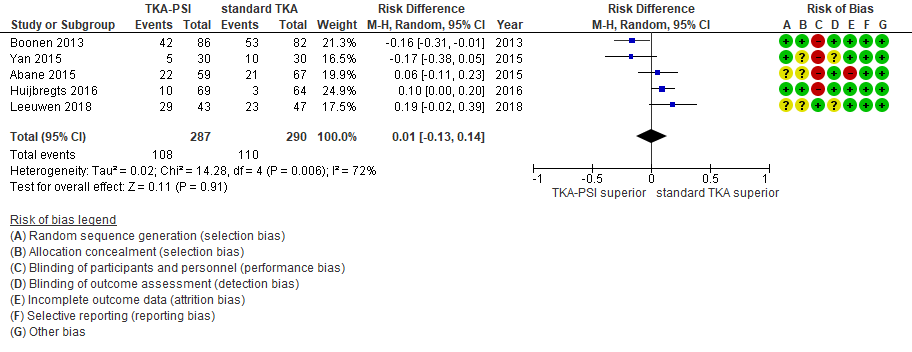


**Appendix I.5: Forest plot in femoral rotational alignment**


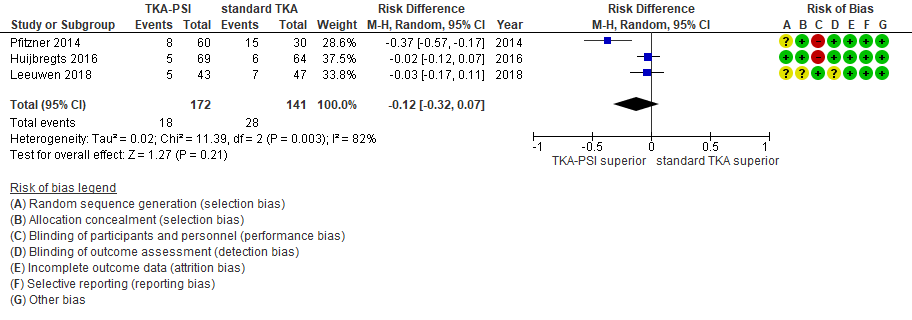


**Appendix I.6: Forest plot in tibial coronal alignment**


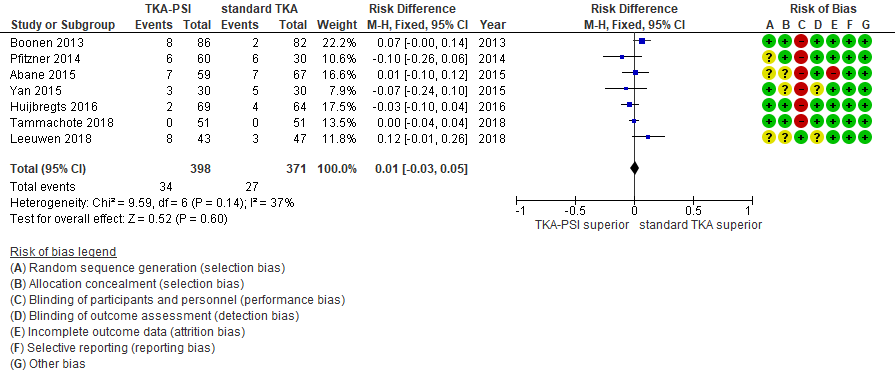


**Appendix I.7: Forest plot in tibial sagittal alignment**


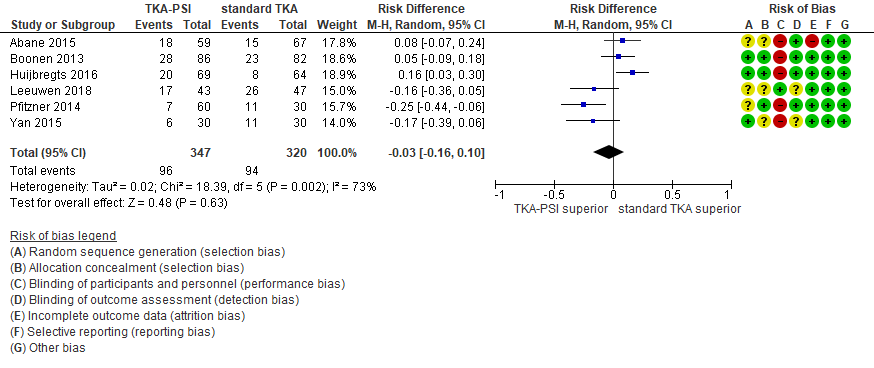


**Appendix J: Funnel plots**

Funnel plot in KSS knee (less than 1-year)


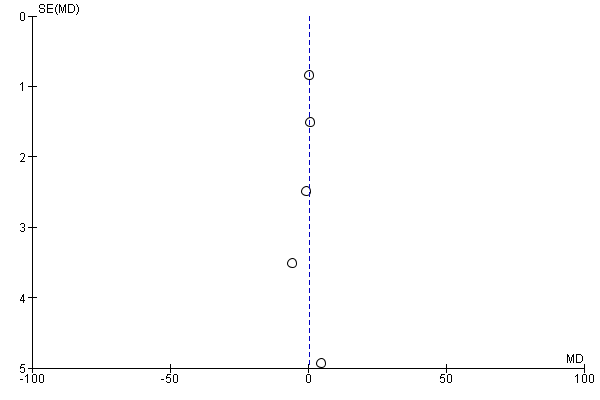


Funnel plot in KSS function (less than 1-year)


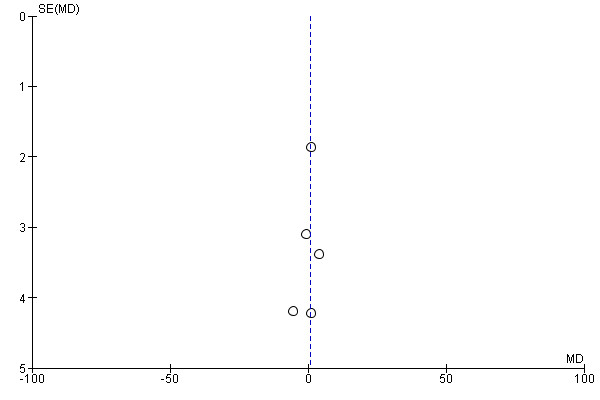


Funnel plot in transfusion rate


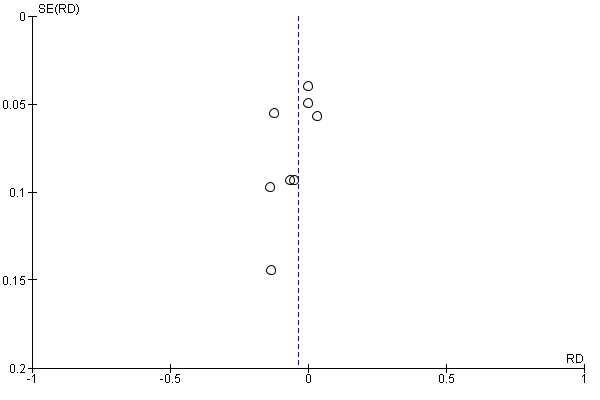


Funnel plot in surgery time


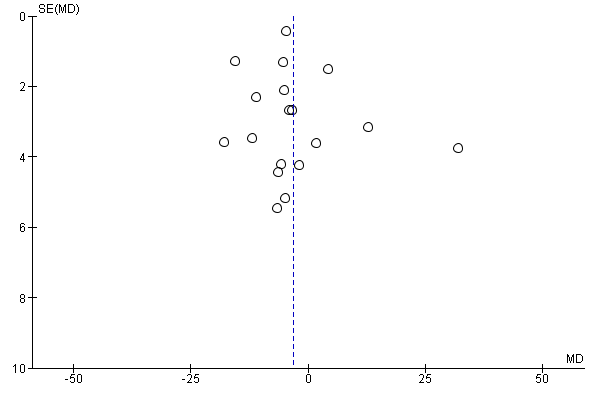


Funnel plot in blood loss


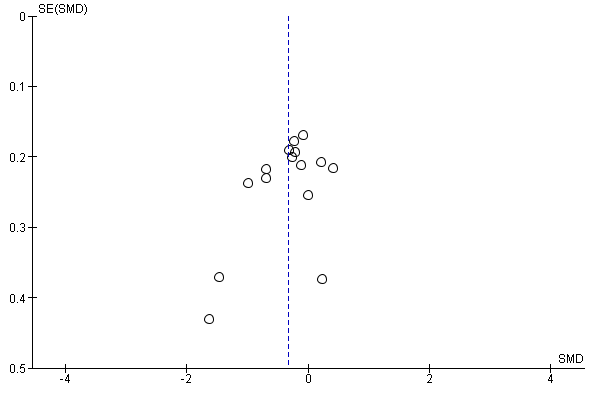


Funnel plot in surgical site infection


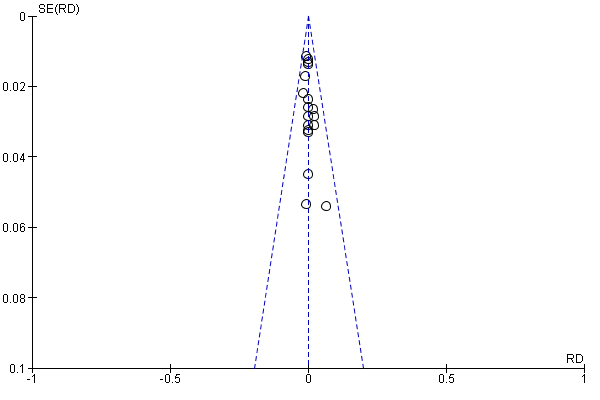


Funnel plot in DVT


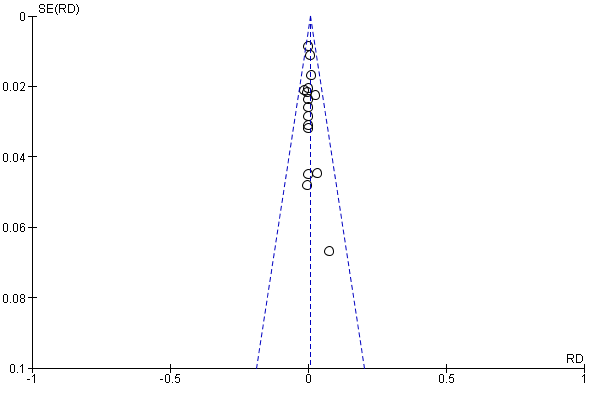


Funnel plot in revision TKA


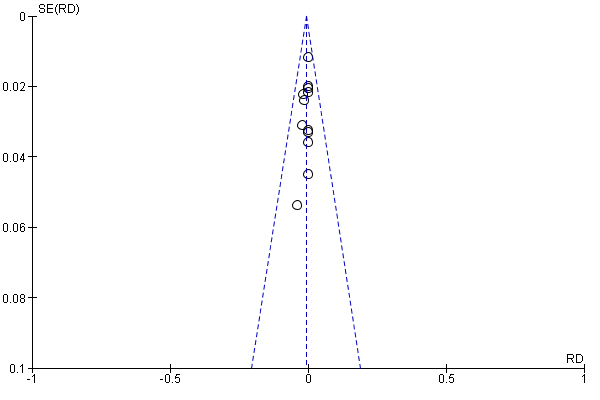


**Appendix K.1: Assessment of the quality of the evidence for Oxford (less than 1-year)**

| **GRADE domains** | **Rating**  **(remember borderline or close calls are possible)** | **Footnotes**  **(explain reasons for rating down)** | **Certainty of the evidence**  **(Circle one)** |
| --- | --- | --- | --- |
| **Risk of Bias**  *(use the Risk of Bias tables and figures)* | Not serious  serious (-1)  very serious (-2) | We could not find specific problems with randomization in the included studies. Blinding was inappropriate in all studies. We downgraded in the domain of risk of bias. | ⊕⊕⊕⊕  High  ⊕⊕⊕🌕  Moderate  ⊕⊕🌕🌕  Low  ⊕🌕🌕🌕  Very Low |
| **Inconsistency** | Not serious  serious (-1)  very serious (-2) | The point estimates were on the same point and the confidence intervals overlapped. The heterogeneity test outputted p-value 0.63 with I^2^=0%. We did not find inconsistency. |  |
| **Indirectness** | Not serious  serious (-1)  very serious (-2) | The included studies with 91.3% weighted recruited osteoarthritis of the knee. Also, the patients were predominantly females and aged over 60. |  |
| **Imprecision** | Not serious  serious (-1)  very serious (-2) | The minimally clinically important difference (MCID) of the Oxford score in total knee arthroplasty is 4.3 (95%CI 3.8-4.8) and the lower/upper limits of the CI in this meta-analysis were less than the MCID. |  |
| **Publication Bias** | Undetected  Strongly suspected (-1) | In the funnel plot, almost all studies were symmetrically distributed, although only four studies were included. |  |
| **Other**  (upgrading factors, circle all that apply) | Large effect (+1 or +2)  Dose response (+1 )  Plausible confounding opposing the effect (+1) | The effect is less significant and there is no plausible reason to rate up. |  |

**Appendix K.2: Assessment of the quality of the evidence for WOMAC (1-year or more)**

| **GRADE domains** | **Rating**  **(remember borderline or close calls are possible)** | **Footnotes**  **(explain reasons for rating down)** | **Certainty of the evidence**  **(Circle one)** |
| --- | --- | --- | --- |
| **Risk of Bias**  *(use the Risk of Bias tables and figures)* | Not serious  serious (-1)  very serious (-2) | We could not find specific problems regarding randomization in the included studies. Blinding was inappropriate in almost all studies with 84.2% weighted, except Dossett 2014. We downgraded in the domain of risk of bias for inappropriate blinding. | ⊕⊕⊕⊕  High  ⊕⊕⊕🌕  Moderate  ⊕⊕🌕🌕  Low  ⊕🌕🌕🌕  Very Low |
| **Inconsistency** | Not serious  serious (-1)  very serious (-2) | The point estimates were almost on the same level and the confidence intervals overlapped, except Dossett 2014. The heterogeneity test presented p-value 0.02 with I^2^=70%. Considering that 84.2% of weighted studies showed the same result, we did not downgrade in the domain of inconsistency. |  |
| **Indirectness** | Not serious  serious (-1)  very serious (-2) | The included studies with 49.2% weighted recruited osteoarthritis of the knee, whereas the other studies with 51.2% weighted recruited patients with knee deformity. Considering that the prominent cause of knee deformity is osteoarthritis and the patients in the included studies were prominently females and patients aged over 60, we did not downgrade. |  |
| **Imprecision** | Not serious  serious (-1)  very serious (-2) | The minimal clinically important difference (MCID) of the WOMAC in knee osteoarthritis is 9.6 (95%CI 5.9 – 12.4) and the lower/upper limits of the CI in this meta-analysis were less than the MCID. |  |
| **Publication Bias** | Undetected  Strongly suspected (-1) | In the funnel plot, almost all studies were symmetrically distributed, although only four studies were included. |  |
| **Other**  (upgrading factors, circle all that apply) | Large effect (+1 or +2)  Dose response (+1)  Plausible confounding opposing the effect (+1) | The effect is less significant and there is no plausible reason to rate up. |  |

**Appendix K.3: Assessment of the quality of the evidence for Surgery time**

| **GRADE domains** | **Rating**  **(remember borderline or close calls are possible)** | **Footnotes**  **(explain reasons for rating down)** | **Certainty of the evidence**  **(Circle one)** |
| --- | --- | --- | --- |
| **Risk of Bias**  *(use the Risk of Bias tables and figures)* | Not serious  serious (-1)  very serious (-2) | We could not find specific problems about randomization in the included studies. In surgical trials, surgeons and scrub nurses could not be blinded (performance bias). Nonetheless, risk of bias was minimized and we did not downgrade in the domain of risk of bias. | ⊕⊕⊕⊕  High  ⊕⊕⊕🌕  Moderate  ⊕⊕🌕🌕  Low  ⊕🌕🌕🌕  Very Low |
| **Inconsistency** | Not serious  serious (-1)  very serious (-2) | The point estimates of the included studies were not on the same level and the confidence intervals did not overlap. The heterogeneity test showed p-value <0.001 with I^2^= 94%. We downgraded in the domain of inconsistency. |  |
| **Indirectness** | Not serious  serious (-1)  very serious (-2) | Overall the included studies recruited patients with osteoarthritis of the knee. Also, they were predominantly female and patients aged over 60, which reflected the general population. We did not downgrade in the domain of indirectness. |  |
| **Imprecision** | Not serious  serious (-1)  very serious (-2) | We pooled 1592 patients in meta-analysis for surgery time. The mean difference in surgery time was 3.09 (95%CI -6.73 – 0.55) and we interpreted that the difference was not clinically important. We did not downgrade in the domain of imprecision. |  |
| **Publication Bias** | Undetected  Strongly suspected (-1) | In the funnel plot, studies were symmetrically distributed. |  |
| **Other**  (upgrading factors, circle all that apply) | Large effect (+1 or +2)  Dose response (+1 )  Plausible confounding opposing the effect (+1) | The effect is less significant and there is no plausible reason to rate up. |  |

**Appendix K.4: Assessment of the quality of the evidence for Blood loss**

| **GRADE domains** | **Rating**  **(remember borderline or close calls are possible)** | **Footnotes**  **(explain reasons for rating down)** | **Certainty of the evidence**  **(Circle one)** |
| --- | --- | --- | --- |
| **Risk of Bias**  *(use the Risk of Bias tables and figures)* | Not serious  serious (-1)  very serious (-2) | We could not find specific problems regarding randomization in the included studies, except Vundelinckx 2013 (6.5% weighted). In surgical trials, surgeons and scrub nurses could not be blinded (performance bias). Nonetheless, risk of bias was minimized and we did not downgrade in the domain of risk of bias. | ⊕⊕⊕⊕  High  ⊕⊕⊕🌕  Moderate  ⊕⊕🌕🌕  Low  ⊕🌕🌕🌕  Very Low |
| **Inconsistency** | Not serious  serious (-1)  very serious (-2) | The point estimates of the included studies were not on the same level and the confidence intervals did not overlap well. The heterogeneity test showed p-value <0.001 with I^2^= 75%. We downgraded in the domain of inconsistency. |  |
| **Indirectness** | Not serious  serious (-1)  very serious (-2) | Overall the included studies recruited patients with osteoarthritis of the knee. Also, they were predominantly female and patients aged over 60, which reflected the general population. We did not downgrade in the domain of indirectness. |  |
| **Imprecision** | Not serious  serious (-1)  very serious (-2) | In meta-analysis, we pooled 1258 patients and the mean difference was a small effect size, which corresponded to 0.4 g/dl (95%CI 0.2-0.9) hemoglobin decrease. The degree of hemoglobin reduction does not clinically influence patients’ health and does not require transfusion. We did not downgrade in the domain of imprecision. |  |
| **Publication Bias** | Undetected  Strongly suspected (-1) | In the funnel plot, studies were symmetrically distributed. |  |
| **Other**  (upgrading factors, circle all that apply) | Large effect (+1 or +2)  Dose response (+1)  Plausible confounding opposing the effect (+1) | The effect is less significant and there is no plausible reason to rate up. |  |

**Appendix K.5: Assessment of the quality of the evidence for transfusion rate**

| **GRADE domains** | **Rating**  **(remember borderline or close calls are possible)** | **Footnotes**  **(explain reasons for rating down)** | **Certainty of the evidence**  **(Circle one)** |
| --- | --- | --- | --- |
| **Risk of Bias**  *(use the Risk of Bias tables and figures)* | Not serious  serious (-1)  very serious (-2) | We could not find specific problems regarding randomization in the included studies, except Vundelinckx 2013 (weighted 6.9%). In surgical trials, surgeons and scrub nurses could not be blinded (performance bias). Nonetheless, risk of bias was minimized and we did not downgrade in the domain of risk of bias. | ⊕⊕⊕⊕  High  ⊕⊕⊕🌕  Moderate  ⊕⊕🌕🌕  Low  ⊕🌕🌕🌕  Very Low |
| **Inconsistency** | Not serious  serious (-1)  very serious (-2) | The point estimates of the included studies were almost on the same level and the confidence intervals overlapped. The heterogeneity test presented p-value 0.28 with I^2^= 19%. We did not downgrade in the domain of inconsistency. |  |
| **Indirectness** | Not serious  serious (-1)  very serious (-2) | Overall the included studies recruited patients with osteoarthritis of the knee. Also, females and age over 60 were predominant in the pooled population, which highly reflected the general population. We did not downgrade in the domain of indirectness. |  |
| **Imprecision** | Not serious  serious (-1)  very serious (-2) | The risk difference was 0.04 (95%CI -0.09 – 0.01, p=0.16). The overall transfusion rate in standard TKA was 20% and the transfusion rate potentially would be shifted to 11% to 21% with wide confidence interval. Also, the pooled events were 98, which is not enough to be confidently conclusive. We downgraded in the domain of imprecision. |  |
| **Publication Bias** | Undetected  Strongly suspected (-1) | In the funnel plot, there was empty area around the right bottom area, suggesting studies with a small sample size might not have been published because of negative results for TKA using PSI. However, the number of included studies was small and the presence of publication bias is debatable. We did not downgrade in the domain of publication bias. |  |
| **Other**  (upgrading factors, circle all that apply) | Large effect (+1 or +2)  Dose response (+1 )  Plausible confounding opposing the effect (+1) | The effect is less significant and there is no plausible reason to rate up. |  |

**Appendix K.6: Assessment of the quality of the evidence for complications**

| **GRADE domains** | **Rating**  **(remember borderline or close calls are possible)** | **Footnotes**  **(explain reasons for rating down)** | **Certainty of the evidence**  **(Circle one)** |
| --- | --- | --- | --- |
| **Risk of Bias**  *(use the Risk of Bias tables and figures)* | Not serious  serious (-1)  very serious (-2) | In non-RCTs, the MINORS score was favorable with low risk of bias. Also, in RCTs, almost all studies properly performed randomization, except Vundelinckx 2013 (weighted 9.0%). Also, overall blinding was properly performed. We did not downgrade in the domain of risk of bias. | ⊕⊕⊕⊕  High  ⊕⊕⊕🌕  Moderate  ⊕⊕🌕🌕  Low  ⊕🌕🌕🌕  Very Low |
| **Inconsistency** | Not serious  serious (-1)  very serious (-2) | The point estimates were almost on the same level and the confidence intervals overlapped. The heterogeneity test accepted the null hypothesis (no heterogenous) with I^2^=0%. We did not downgrade in the domain of inconsistency. |  |
| **Indirectness** | Not serious  serious (-1)  very serious (-2) | Overall the included studies recruited patients with osteoarthritis of the knee. Also, they were predominantly females and patients aged over 60. They reflected the general population. We did not downgrade in the domain of indirectness. |  |
| **Imprecision** | Not serious  serious (-1)  very serious (-2) | The total events in the composite outcome were 44. The complication rate in standard TKA was 1.7%. The risk difference was 0.00 (95%CI -0.01 – 0.01) and the complication rates would shift from 0.7% to 2.7%. We interpreted the confidence interval was not narrow and we downgraded in the domain of imprecision. |  |
| **Publication Bias** | Undetected  Strongly suspected (-1) | In the funnel plot, studies were symmetrically distributed. |  |
| **Other**  (upgrading factors, circle all that apply) | Large effect (+1 or +2)  Dose response (+1 )  Plausible confounding opposing the effect (+1) | The effect is less significant and there is no plausible reason to rate up. |  |
